# Comparison of freshly cultured versus cryopreserved mesenchymal stem cells in animal models of inflammation: A pre-clinical systematic review

**DOI:** 10.7554/eLife.75053

**Published:** 2022-07-15

**Authors:** Chintan Dave, Shirley HJ Mei, Andrea McRae, Christine Hum, Katrina J Sullivan, Josee Champagne, Tim Ramsay, Lauralyn McIntyre

**Affiliations:** 1 Division of Critical Care Medicine, Department of Medicine, Western University London Canada; 2 Regenerative Medicine Program, Ottawa Hospital Research Institute Ottawa Canada; 3 Knowledge Synthesis Group, Ottawa Hospital Research Institute Ottawa Canada; 4 University of Ottawa Ottawa Canada; 5 Clinical Epidemiology, Ottawa Hospital Research Institute Ottawa Canada; 6 Division of Critical Care, Department of Medicine, University of Ottawa Ottawa Canada; University of Cambridge United Kingdom; Icahn School of Medicine at Mount Sinai United States

**Keywords:** cryopreserved, mesenchymal stem cells, inflammation, freshly-cultured, systematic review, Other

## Abstract

**Background:**

Mesenchymal stem cells (MSCs) are multipotent cells that demonstrate therapeutic potential for the treatment of acute and chronic inflammatory-mediated conditions. Although controversial, some studies suggest that MSCs may lose their functionality with cryopreservation which could render them non-efficacious. Hence, we conducted a systematic review of comparative pre-clinical models of inflammation to determine if there are differences in in vivo measures of pre-clinical efficacy (primary outcomes) and in vitro potency (secondary outcomes) between freshly cultured and cryopreserved MSCs.

**Methods:**

A systematic search on OvidMEDLINE, EMBASE, BIOSIS, and Web of Science (until January 13, 2022) was conducted. The primary outcome included measures of in vivo pre-clinical efficacy; secondary outcomes included measures of in vitro MSC potency. Risk of bias was assessed by the SYRCLE ‘Risk of Bias’ assessment tool for pre-clinical studies.

**Results:**

Eighteen studies were included. A total of 257 in vivo pre-clinical efficacy experiments represented 101 distinct outcome measures. Of these outcomes, 2.3% (6/257) were significantly different at the 0.05 level or less; 2 favoured freshly cultured and 4 favoured cryopreserved MSCs. A total of 68 in vitro experiments represented 32 different potency measures; 13% (9/68) of the experiments were significantly different at the 0.05 level or less, with seven experiments favouring freshly cultured MSC and two favouring cryopreserved MSCs.

**Conclusions:**

The majority of preclinical primary in vivo efficacy and secondary in vitro potency outcomes were not significantly different (p<0.05) between freshly cultured and cryopreserved MSCs. Our systematic summary of the current evidence base may provide MSC basic and clinical research scientists additional rationale for considering a cryopreserved MSC product in their pre-clinical studies and clinical trials as well as help identify research gaps and guide future related research.

**Funding:**

Ontario Institute for Regenerative Medicine

## Introduction

Mesenchymal stromal cells (mesenchymal stem cells; MSCs) are multipotent stem cells that can be isolated from many adult tissues (e.g. bone marrow, adipose tissue) (Pittenger et al., 2019). MSCs have been studied in clinical trials for almost two decades (Koç et al., 2000), and have since been implicated in use for diverse conditions (Gomez-Salazar et al., 2020). MSCs release growth factors and cytokines along with extracellular vesicles to activate cell proliferation, prevent apoptosis, and ultimately improve regenerative response (Pittenger et al., 2019). MSCs may also modulate the immune response by decreasing inflammation, reducing scar formation, increasing pathogen clearance, altering endothelial permeability, and improving mitochondrial dysfunction as demonstrated in different pre-clinical models of inflammation (Fish and Hajjar, 2015; Hoogduijn et al., 2010; Gupta et al., 2012; Islam et al., 2012; Li et al., 2018; Tsubokawa et al., 2010). The mechanism for how MSCs modulate inflammation and promote healing is not yet completely understood; however, the observed effect may be mediated by both the direct contact with immune cells and release of soluble factors (Caplan, 2009; Shi et al., 2012; Souza-Moreira et al., 2022). Given their potent immunomodulatory effects, MSCs are particularly attractive for use in infectious as well as acute and chronic inflammatory conditions. There are a growing number of studies that demonstrate the efficacy of MSC therapy in a variety of pre-clinical models, such as acute lung injury (Chang et al., 2014; Mei et al., 2007; Matthay et al., 2010; Weiss et al., 2013; Wilson et al., 2015), sepsis (McIntyre et al., 2018; Mei et al., 2010), acute myocardial infarction (Boyle et al., 2010), multiple sclerosis (Connick et al., 2011), graft-versus-host disease (Baron et al., 2010; Introna et al., 2014; Pérez-Simon et al., 2011), osteoarthritis (Emadedin et al., 2015; Jo et al., 2014; Orozco et al., 2014; Vega et al., 2015; Vives et al., 2015), and inflammatory bowel disease (IBD) (Forbes et al., 2014; Molendijk et al., 2015). Moreover, as of March 10, 2022, 1,097 active trials involving MSCs were registered (https://www.clinicaltrials.gov). Although MSCs have potential to treat many clinical conditions, a major limitation with nearly all studies is the constrained real-world applicability, where it is vital to have an intervention that is readily available and administered in a time-sensitive manner. For this to occur, the MSCs must overcome the logistical challenges of in-vitro isolation and culture, effective cryopreservation methodology, and a route for rapid accessibility to the bedside. Future real-world therapeutic applications of MSCs will need to be ready for immediate use as off-the-shelf products in urgent medical situations (Mendicino et al., 2014; Woods et al., 2016).

To date, a majority of preclinical MSC research employ freshly cultured MSCs. In a recent systematic review of the safety of MSCs in 55 randomized clinical trials, only 15 (27%) used cryopreserved cells (Thompson et al., 2020), potentially due to the concern that cryopreserved MSCs may lose some of their functionality (Galipeau et al., 2016). Some in vitro studies demonstrate a negative impact of cryopreservation on MSC function (François et al., 2012; Chinnadurai et al., 2016); however, others suggest that cryopreservation may not negatively impact their functionality (Cruz et al., 2015; Devaney et al., 2015; Gramlich et al., 2016; Luetzkendorf et al., 2015).

To evaluate evidence currently available in the literature, our team conducted a systematic synthesis of all pre-clinical comparative studies that examined freshly cultured versus cryopreserved MSCs on surrogate measures of in vivo efficacy (primary outcomes) and in vitro potency (secondary outcomes) in animal models of inflammation. The protocol for our systematic review is published in *Systematic Reviews* (https://doi.org/10.1186/s13643-020-01437-z) and registered in PROSPERO (CRD42020145833).

## Materials and methods

### Search strategy

We conducted electronic search strategies without language restriction of Ovid platform, Ovid MEDLINE, OvidMEDLINE In-Process & Other Non-Indexed Citations, Embase Classic plus Embase, and BIOSIS and Web of Science using Web of Knowledge until January 13, 2022. Given the non-standard terminology associated with MSCs, several pre-defined terms were used, and the electronic and manual search strategies were developed and tested through an iterative process by an experienced medical information specialist in consultation with the research team (Supplementary file 1). Six target articles provided by an expert in the field of preclinical research (SM) that were known prior to the search were included in the search criteria to help capture all potential studies. No additional filters were employed to ensure the largest number of relevant studies are captured. We followed the PRISMA guidelines (Supplementary file 2) for reporting our systematic review.

### Assessment of risk of bias

Risk of bias was assessed independently by two reviewers (CD and AM), and disagreements were resolved via consensus, or by a third reviewer when necessary. All studies were assessed as high, low, or unclear for the 10 domains of bias adapted from the SYRCLE ‘Risk of Bias’ assessment tool for pre-clinical in vivo studies (Hooijmans et al., 2014). This tool has been adapted from the Cochrane Collaboration Risk of Bias tool employed in clinical studies, with an aim to incorporate key elements that are relevant for in vivo animal studies. The prompting questions employed to assess risk of bias (AGREE tool) can be found in Supplementary file 3. The 10 risk-of-bias domains and signalling questions are provided in Table 7***.***

### Study eligibility

Pre-clinical studies of in vivo models of inflammation that directly compared freshly cultured to cryopreserved MSC products (randomized, quasi-randomized, and non-randomized designs) were included. To be defined as cryopreserved, MSCs could have been cryopreserved for any duration of time and/or be placed in culture for less than 24 hr post-thaw prior to use in the given experiment. MSCs were considered freshly cultured when the cells were either in continuous culture or cryopreserved but then thawed and placed in culture for at least 24 hr prior to use in experiments. We used this 24-hr culture time as a cut-off as previous experiments suggest that cryopreserved MSCs may require 24 hr of culture to recover their functionality (Galipeau, 2013). The study must have included an *animal* model of inflammation where the intervention and comparison groups examined the administration of cryopreserved and freshly cultured MSCs, respectively, delivered by any route, and derived from the same MSC origin (ex. bone marrow, adipose tissue, umbilical cord, or other) and source (xenogeneic, syngeneic, autologous, or allogeneic). MSCs that were pre-treated, pre-conditioned, genetically altered, or co-administered with other experimental interventions were included if the same alteration was applied to both the freshly cultured and cryopreserved MSCs.

Studies that administered MSCs before or during the induction of the experimental pre-clinical model (i.e. prevention studies) were excluded. We also excluded studies of immunocompromised animals (SCID) or treatments to immunosuppress the animals were excluded because our primary aim was to examine the efficacy of cryopreserved versus freshly cultured MSCs on measures of inflammation in animal models with an intact immune system. Moreover, an intact immune system may be required for MSC immunomodulation via the host cytotoxic cell activity (Galleu et al., 2017). Studies that examined the effects of MSCs on implantation and tissue regeneration (e.g. bone regeneration), or compared differentiated MSCs (e.g. differentiated into a myocyte), Mesenchymal Progenitor Cells (MPCs), Mononuclear Cell (MNC) fraction, or stem cells that were not described as MSCs, and studies that only reported in vitro experiments comparing freshly cultured to cryopreserved MSC products were also excluded.

### Outcomes

The primary outcomes were surrogate measures of in vivo pre-clinical efficacy that were relevant to specific acute and chronic inflammatory animal models and defined by two outcome domains: 1) The Function and Composition of Tissues (e.g. organ dysfunction, histopathological damage); and 2) Protein Expression and Secretion (e.g. cytokine levels, immunohistochemistry analysis).

Secondary outcomes included measures of in vitro MSC potency (that were described as additional experiments in the included in vivo studies). Ideally, potency should represent the MSCs’ mechanism of action; however, MSCs have complex and multiple mechanisms of action, all of which are not yet fully characterized or reported (Galipeau et al., 2016). In accordance with the International Society for Cellular Therapy perspective paper on this topic (Galipeau et al., 2016), MSC potency was based on an assay matrix (collection of assays) that included a combination of in vitro analytical and/or biological assays (e.g. the cellular secretome by ELISA [enzyme-linked immunosorbent assay], or functional cell-based assays [in vitro assay culturing MSCs with responder immune cells] respectively). Hence, the two main secondary in vitro potency outcome domains were: 1) Co-culture assays; and 2) Protein Expression and Secretion (ex: cytokine levels).

### Study selection and data collection

The titles and abstracts were screened independently by two members (CD, ED). The full-text of all potentially eligible studies were retrieved and reviewed for eligibility, independently, by two members of the team using the a priori eligibility criteria described above. Disagreements between reviewers were resolved by consensus or by a third member of the systematic review team (LM, SM). Data were extracted independently by two members of the research team into standardized, pilot-tested excel sheet forms (Supplementary file 4). Authors were contacted for data clarification or for additional data when required.

### Data analysis

Meta-analyses were planned as per protocol, if sufficient data were available and if appropriate: two or more studies with similar disease models for an in vivo pre-clinical efficacy outcome, with the same outcome definition. Data reported in non-standard format (e.g. mean ± standard error, median and range) was converted to mean ± standard deviation. Given the complexity and variety of results, the results were summarized in tabular format and presented as number of experiments that reached statistical significance at the 0.05 level.

## Results

### Search results and study characteristics

**Figure 1.**
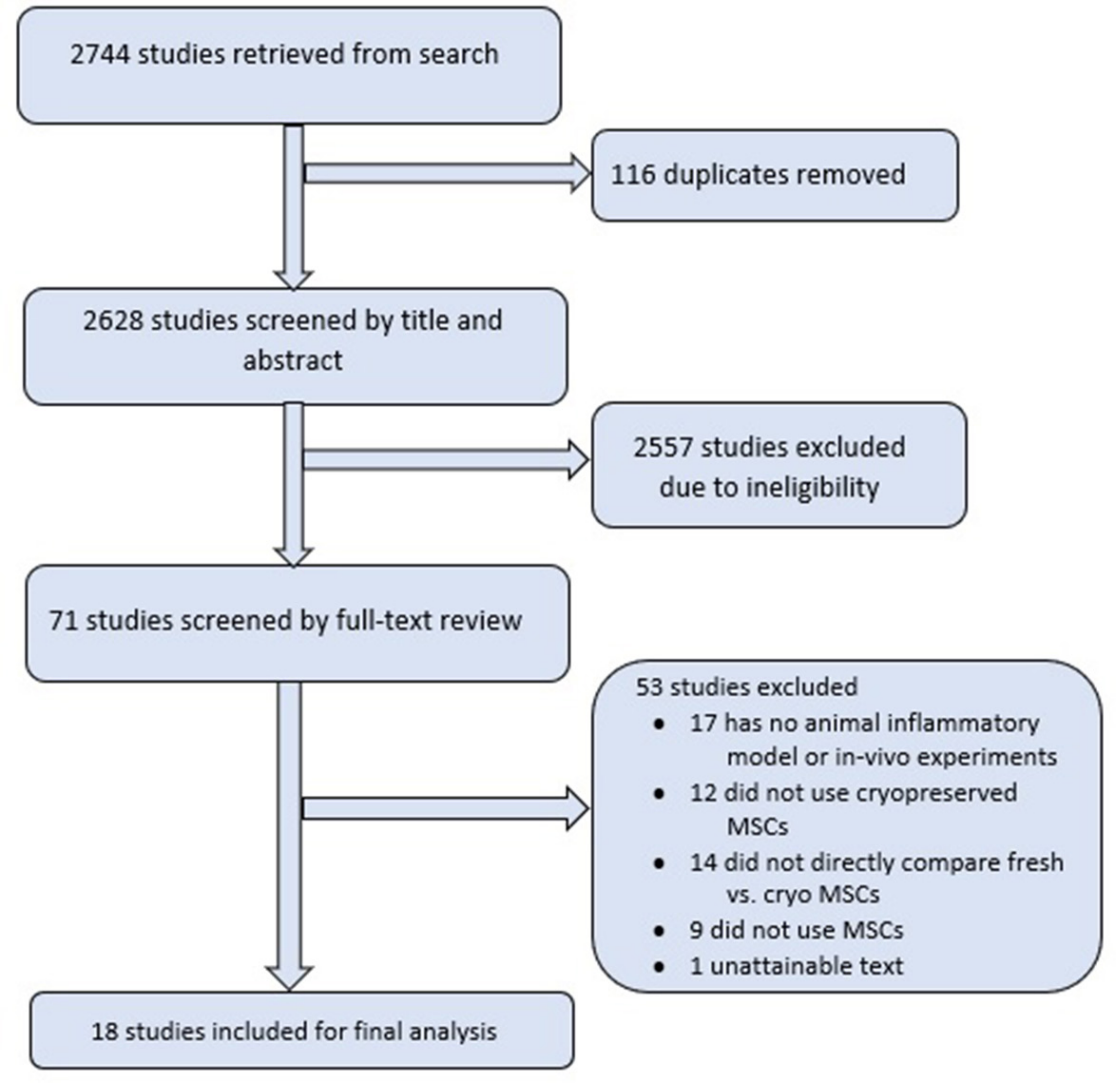
Literature search and study inclusion.

The search strategy yielded 2744 potential studies; and after applying the eligibility criteria and full text review, 18 studies were deemed eligible for inclusion (Figure 1; Cruz et al., 2015; Devaney et al., 2015; Gramlich et al., 2016; Salmenkari et al., 2019; Somal et al., 2017; Tan et al., 2019; Curley et al., 2017; Horiuchi et al., 2021; Horie et al., 2021; Yea et al., 2020; Bárcia et al., 2017; Bharti et al., 2020; Khan et al., 2019; Horie et al., 2020a; Lohan et al., 2018; Perlee et al., 2019; Rogulska et al., 2019).

Eight studies used mice for their experiments (Cruz et al., 2015; Gramlich et al., 2016; Salmenkari et al., 2019; Somal et al., 2017; Tan et al., 2019; Curley et al., 2017; Perlee et al., 2019; Rogulska et al., 2019), seven studies used rats (Devaney et al., 2015; Curley et al., 2017; Horiuchi et al., 2021; Horie et al., 2021; Yea et al., 2020; Lohan et al., 2018; Horie et al., 2020b), one study used both mice and rats (Bárcia et al., 2017), one study used beagle dogs (Khan et al., 2019), and one study used guinea pigs (Bharti et al., 2020). Twelve studies included a ’vehicle only’ as an additional control arm (Devaney et al., 2015; Gramlich et al., 2016; Salmenkari et al., 2019; Somal et al., 2017; Horiuchi et al., 2021; Horie et al., 2021; Yea et al., 2020; Bharti et al., 2020; Lohan et al., 2018; Perlee et al., 2019; Rogulska et al., 2019; Horie et al., 2020b), while four studies employed a sham animal model, where disease negative animals received MSCs or vehicle (Tan et al., 2019; Curley et al., 2017; Bárcia et al., 2017; Horie et al., 2020a). One study directly compared cryopreserved and freshly cultured MSCs without an additional control arm (Khan et al., 2019) and one study employed a sham model, vehicle, and cryopreserved and freshly cultured fibroblasts as controls (Cruz et al., 2015).

Of the 18 included studies, seven studied models of preclinical lung injury and sepsis (Devaney et al., 2015; Tan et al., 2019; Curley et al., 2017; Horie et al., 2021; Horie et al., 2020a; Perlee et al., 2019; Horie et al., 2020b), four a wound healing model (Somal et al., 2017; Yea et al., 2020; Bharti et al., 2020; Rogulska et al., 2019), three of neurological or ocular disease, specifically one of corneal transplantation (Lohan et al., 2018), retinal ischemia/reperfusion (Gramlich et al., 2016), and spinal cord injury model (Khan et al., 2019), and one each of allergic airway inflammatory disease (Cruz et al., 2015), wound healing and chronic inflammatory arthritis (Bárcia et al., 2017), acute and chronic inflammatory colitis (Salmenkari et al., 2019), and chronic osteoarthritis (Horiuchi et al., 2021). Complete reporting of inflammatory models, MSC origins and characteristics can be found in Tables 1 and 2.

**Table 1.** Models of inflammation and characteristics of included studies.

First Author (Year)	Animal Inflammatory Model	Country	Language of Publication	Species	Strain	Gender	Sample size	Age (range)	Weight (grams)
Bárcia et al., 2017	1) Chronic adjuvant-induced arthritis (AIA) model 2) Hindlimb ischemia model	Portugal	English	1) Rat 2) Mouse	1) Winstar 2) C57BL/6	1) Male 2) Female	1) 18 2) 36	1) NR 2) 12 weeks	1) 365–480 g 2) NR
Cruz et al., 2015	Allergic Airways Inflammation induced by Aspergillus hyphal extract (AHE) exposure in immunocompetent mice	USA	English	Mouse	C57BL/6	Male	72	8–12 weeks	NR
Curley et al., 2017	Acute respiratory distress syndrome by intratracheal instillation of *E. coli*	Canada	English	Rat	Sprague-Dawley (specific pathogen-free)	Male	NR	NR	350–450 g
Devaney et al., 2015	Acute lung injury induced by *E. coli* pneumonia	Ireland	English	Rat	Sprague-Dawley (specific pathogen-free)	Male	40	NR	350–450 g
Gramlich et al., 2016	Retinal Ischemia/Reperfusion Injury Model	USA	English	Mouse	C57BL6/J	Male and Female	37	2 months	NR
Lohan et al., 2018	Corneal Transplantation	Ireland	English	Rat	Lewis	Male	NR	8–14 weeks	NR
Salmenkari et al., 2019	Colitis (3% DSS)	Finland	English	Mouse	Balb/c	Male	NR	8 weeks	NR
Somal et al., 2017	Wound healing	India	English	Rat	Wistar	Male	27	NR	NR
Bharti et al., 2020	Wound healing	India	English	Guinea pigs	Dunkin Hartley	Male	25	NR	NR
Horie et al., 2020a	Ventilator-induced Lung Injury	Ireland	English	Rats	NR	NR	NR	NR	NR
Horie et al., 2020a	*E. coli*-induced lung injury	Ireland	English	Rats	Pathogen-free sprague Dawley	Male	NR	NR	300–450 g
Khan et al., 2019	Spinal Cord Injury induced through a balloon compression method	Korea	English	Dog	Beagle	NR	12	1.2+/-0.2 years	12+/-3 kg
Rogulska et al., 2019	Wound healing	Ukraine	English	Mouse	Balb/C	Male	27	5–6 months	25–30 g
Tan et al., 2019	Polymicrobial sepsis induced by cecal-ligation-and-puncture (CLP)	Canada	English	Mouse	C57BL6/J	Female	NR	8 weeks	17–21 g
Perlee et al., 2019	K.K. pneumoniae induced pneumosepsis	Netherlands	English	Mouse	Pathogen free C57BL/6	Female	NR	8–10 weeks	NR
Yea et al., 2020	Wound healing	Korea	English	Rat	Sprague-Dawley	Male	120	12 weeks	340–360 g
Horiuchi et al., 2021	Osteoarthritis	Japan	English	Rat	Wildtype Lewis	Female	40	10 weeks	180–200 g
Horie et al., 2021	Ventilator-Induced Lung Injury	Ireland	English	Rat	Sprague-Dawley	Male	28	NR	350–450 g

NR = Not Reported.

**Table 2.** MSC characteristics of included studies.

First author (Year)	Species and tissue source	Compatibility with animal	ISCT criteria met	Route of administration	Vehicle	Timing of MSCs post-disease induction	Fresh MSCs		Cryopreserved MSCs		
							Cryopreserved at any point?	Duration of culture	Method	Duration	Time from Thaw to Experiment
Bárcia et al., 2017	Human Umbilical Cord	Xenogenic	Yes	1) Intra-articular 2) Intra-muscular	PBS	1) 7 days 2) 5 hr	No	>5 days	Controlled Rate Freezer	NR	Immediately
Cruz et al., 2015	Human and Murine Bone Marrow	Syngenic and Xenogenic	Yes	Intravenous	PBS	14 days	Yes	NR	-–80°C for 48 hr then liquid nitrogen	9 days	15 min
Curley et al., 2017	Human Umbilical Cord and Bone Marrow	Xenogenic	Yes	Intravenous	PBS	NR	No	4 days	Controlled Rate Freezer	NR	Day of administration
Devaney et al., 2015	Human Bone Marrow	Xenogenic	Yes	Intravenous	PBS	0.5 hr	Yes	NR	NR	NR	30 min
Gramlich et al., 2016	Human	Xenogenic	Yes	Intra-ocular	PBS	2 hr	Yes	>7 days	Controlled Rate Freezer	7–30 days	<1 hr
Lohan et al., 2018	Rat Bone Marrow	Allogenic	NR	Intravenous	PBS	1 and 7 days prior	Yes	NR	–80°C for 24 hr then liquid nitrogen	NR	Immediately
Salmenkari et al., 2019	Human Bone Marrow	Xenogenic	NR	Intravenous	0.9% NaCl +3.6% HAS	3 and 5 days	Yes	NR	NR	NR	NR
Somal et al., 2017	Gravid caprine AF (amniotic fluid), AS (amniotic sac), WJ (Wharton's jelly), CB (cord blood)	Xenogenic	NR	Subcutaneously	PBS	7, 14, 21, 28 days	Yes	NR	–80°C overnight then liquid nitrogen	Atleast 1 month	NR
Bharti et al., 2020	Dog Bone Marrow	Xenogenic	NR	Surgically placed over wound	Polypropylene mesh	NR	Yes	NR	–80°C overnight then liquid nitrogen	1 month	NR
Horie et al., 2020a	Human Bone Marrow	Xenogenic	NR	Intravenous	PBS	6 hr	Yes	NR	NR	NR	NR
Horie et al., 2020a	Human Bone Marrow and Umbilical Cord	Xenogenic	NR	Intra-tracheal	PBS	30 min	Yes	NR	NR	NR	Immediately
Khan et al., 2019	Dog Adipose Tissue	Allogenic	NR	Intravenous	Hartmann’s Solution	Immediately	Yes	NR	4 °C for 1 hr, –20 °C for 2 hr, –80 °C for 24 hr, then –150 °C	2–3 weeks	Immediately
Rogulska et al., 2019	Human Adipose Tissue	Xenogenic	NR	Implantation into wound	3D gel	Immediately	Yes	NR	–80°C the liquid nitrogen	1 month	NR
Tan et al., 2019	Human Bone Marrow	Xenogenic	Yes	Intravenous	5% Human Albumin in PlasmaLyte	6 hr	No	>24 hr	Controlled Rate Freezer	NR	Immediately
Perlee et al., 2019	Human Adipose Tissue	Xenogenic	Yes	Intravenous	Ringer’s Lactate	1 or 6 hr	No	24 hr	Liquid nitrogen	Until required	Day of administration
Yea et al., 2020	Human Umbilical Cord	Xenogenic	NR	Intratendinous	PBS	Immediately	No	NR	–80°C then –196 °C Liquid Nitrogen	Up to 1 month	Immediately
Horiuchi et al., 2021	Rat Synovial Fluid	Allogenic	NR	Intraarticular	PBS	Every week from 2 to 8 weeks	Yes	7 days	–80 °C overnight, and then at –150 °C	16 months	Immediately
Horie et al., 2021	Human Umbilical Cord	Xenogenic	NR	Intravenous	PBS	15 min	No	NR	NR	Up to 2 months	Immediately

### Description of cryopreservation and thaw process for cryopreserved MSCs

The duration of cryopreservation for cryopreserved MSCs prior to use in experiments was not reported in nine studies (Devaney et al., 2015; Salmenkari et al., 2019; Tan et al., 2019; Curley et al., 2017; Bárcia et al., 2017; Horie et al., 2020a; Lohan et al., 2018; Perlee et al., 2019; Horie et al., 2020b), four studies cryopreserved the MSCs for at least 1 month (Somal et al., 2017; Horiuchi et al., 2021; Bharti et al., 2020; Rogulska et al., 2019), and two for up to 2 months (Horie et al., 2021; Yea et al., 2020). One study cryopreserved MSCs for 2–3 weeks (Khan et al., 2019), another between 1 and 4 weeks (Gramlich et al., 2016), and one study cryopreserved their MSCs for 9 days (Cruz et al., 2015).

Ten studies used 10% DMSO (dimethyl sulfoxide) as part of their cryopreservation solution (Cruz et al., 2015; Devaney et al., 2015; Salmenkari et al., 2019; Somal et al., 2017; Yea et al., 2020; Bárcia et al., 2017; Bharti et al., 2020; Khan et al., 2019; Lohan et al., 2018; Rogulska et al., 2019), three studies used CryoStor Cell Preservation Media (Sigma-Aldrich) (Gramlich et al., 2016; Horie et al., 2021; Horie et al., 2020a), one study used MSC Freezing media (Biological Industries) (Tan et al., 2019), one study used 5% DMSO (Horiuchi et al., 2021), and three studies did not report the solution used for cryopreservation (Curley et al., 2017; Perlee et al., 2019; Horie et al., 2020b). Five studies did not report on their method of cryopreservation (Devaney et al., 2015; Salmenkari et al., 2019; Horie et al., 2021; Horie et al., 2020a; Horie et al., 2020b), three studies employed a controlled-rate freezer to achieve cryopreservation (Tan et al., 2019; Curley et al., 2017; Bárcia et al., 2017), while eight studies used liquid nitrogen at –80°C to –196°C (Cruz et al., 2015; Gramlich et al., 2016; Somal et al., 2017; Yea et al., 2020; Bharti et al., 2020; Lohan et al., 2018; Perlee et al., 2019; Rogulska et al., 2019) for storage, and two studies gradually cryopreserved the MSCs with decremental temperature over 24 hr, followed by storage at –150 °C (Horiuchi et al., 2021; Khan et al., 2019).

Eight studies did not report their thawing protocol (Cruz et al., 2015; Devaney et al., 2015; Somal et al., 2017; Horie et al., 2021; Bárcia et al., 2017; Bharti et al., 2020; Horie et al., 2020a; Perlee et al., 2019), one study employed a cell-thawing device called the ThawStar (AsteroBio, USA) (Horiuchi et al., 2021) and the remaining nine studies used a 37 °C hot water bath to thaw the cryopreserved MSCs (Gramlich et al., 2016; Salmenkari et al., 2019; Tan et al., 2019; Curley et al., 2017; Yea et al., 2020; Khan et al., 2019; Lohan et al., 2018; Rogulska et al., 2019; Horie et al., 2020b). Two studies thawed MSCs on the day of administration for their experiments (Curley et al., 2017; Perlee et al., 2019), while nine studies reported thawing MSCs either immediately or within 1 hr of use in experimentation (Cruz et al., 2015; Devaney et al., 2015; Gramlich et al., 2016; Tan et al., 2019; Yea et al., 2020; Bárcia et al., 2017; Khan et al., 2019; Lohan et al., 2018; Horie et al., 2020b). Seven studies did not report time from thaw to use in experimentation (Salmenkari et al., 2019; Somal et al., 2017; Horiuchi et al., 2021; Horie et al., 2021; Bharti et al., 2020; Horie et al., 2020a; Rogulska et al., 2019). Nine studies suspended thawed MSCs in phosphate buffered saline (PBS, vehicle for experiments) (Cruz et al., 2015; Devaney et al., 2015; Tan et al., 2019; Curley et al., 2017; Horiuchi et al., 2021; Bárcia et al., 2017; Khan et al., 2019; Lohan et al., 2018; Horie et al., 2020b), while one study re-suspended them in ringer’s lactate supplemented with 3% Dimethyl sulfoxide (DMSO) (Perlee et al., 2019), one used MSCs suspended in 0.9% NaCl +3.6% HSA (Human Serum Albumin) (Salmenkari et al., 2019), one used PBS with 5% HSA (Tan et al., 2019), and six studies did not report their resuspension solution (Horie et al., 2021; Yea et al., 2020; Bharti et al., 2020; Horie et al., 2020a; Lohan et al., 2018; Rogulska et al., 2019).

### Description of cryopreservation and culture process for freshly cultured MSCs

Freshly cultured MSCs were not cryopreserved at any point after harvest from source in 13 studies (range of total culture time: 4–28 days) (Cruz et al., 2015; Devaney et al., 2015; Gramlich et al., 2016; Salmenkari et al., 2019; Somal et al., 2017; Horie et al., 2021; Yea et al., 2020; Bharti et al., 2020; Khan et al., 2019; Horie et al., 2020a; Lohan et al., 2018; Rogulska et al., 2019; Horie et al., 2020b). In five studies, the MSCs were cryopreserved and then culture-expanded for more than 24 hr prior to use in experimentation (Tan et al., 2019; Curley et al., 2017; Horiuchi et al., 2021; Bárcia et al., 2017; Perlee et al., 2019).

Further details related to MSC culture, including medium, passage, concentration, and route of administration can be found in Table 2.

### Risk of bias

Of the 18 included studies, none of them met low-risk of bias criteria for all 10 domains and all studies demonstrated unclear risk of bias due to lack or reporting in atleast two domains. Ten studies did not have any features that would confer a high-risk of bias in the one of the 10 domains (Cruz et al., 2015; Devaney et al., 2015; Tan et al., 2019; Curley et al., 2017; Horiuchi et al., 2021; Horie et al., 2021; Yea et al., 2020; Bharti et al., 2020; Khan et al., 2019; Horie et al., 2020a). Five studies demonstrated high-risk of bias in one domain (Devaney et al., 2015; Salmenkari et al., 2019; Somal et al., 2017; Perlee et al., 2019; Rogulska et al., 2019), and the remaining three studies demonstrated high-risk of bias in two or more domains (Gramlich et al., 2016; Bárcia et al., 2017; Lohan et al., 2018). The complete reporting of the risk of bias domains is presented in Table 3.

**Table 3.** Risk of Bias assessments for the included in vivo studies using SYRCLE Tool.

*Selection Bias*				*Performance Bias*		*Detection Bias*		*Attrition Bias*	*Reporting Bias*	*Other Bias*
**Author (year)**	**Adequate randomization**	**Baseline charactersics given**	**Evidence of adequate concealment of groups**	**Evidence of random housing of animals**	**Evidence of caregivers blinded to intervention**	**Evidence of random selection for assessment**	**Evidence of assessor blinded**	**Explanation of missing animal data**	**Free of selective reporting based on methods/results**	**Free of other high bias risk**
Bárcia et al., 2017	Unclear	Yes (Low Risk)	Unclear	Yes (Low Risk)	No (High Risk)	Unclear	No (High Risk)	Yes (Low Risk)	Yes (Low Risk)	No (High Risk)
Bharti et al., 2020	Unclear	Unclear	Unclear	Yes (Low Risk)	Unclear	Unclear	Unclear	Unclear	Yes (Low Risk)	Yes (Low Risk)
Cruz et al., 2015	Unclear	Yes (Low Risk)	Unclear	Yes (Low Risk)	Unclear	Unclear	Yes (Low Risk)	Unclear	Yes (Low Risk)	Yes (Low Risk)
Curley et al., 2017	Unclear	Yes (Low Risk)	Unclear	Unclear	Unclear	Unclear	Yes (Low Risk)	Unclear	Yes (Low Risk)	Yes (Low Risk)
Devaney et al., 2015	Unclear	Yes (Low Risk)	Unclear	Unclear	Unclear	Unclear	No (High Risk)	Yes (Low Risk)	Yes (Low Risk)	Yes (Low Risk)
Gramlich et al., 2016	No (High Risk)	Yes (Low Risk)	Unclear	Unclear	Unclear	Unclear	Yes (Low Risk)	Unclear	Yes (Low Risk)	No (High Risk)
Horie et al., 2020a	Unclear	Unclear	Unclear	Unclear	Unclear	Unclear	Yes (Low Risk)	Unclear	Yes (Low Risk)	Yes (Low Risk)
Horie et al., 2020a	Unclear	Unclear	Unclear	Unclear	Unclear	Unclear	Unclear	Yes (Low Risk)	Yes (Low Risk)	Yes (Low Risk)
Khan et al., 2019	Unclear	Yes (Low Risk)	Unclear	Unclear	Yes (Low Risk)	Unclear	Yes (Low Risk)	Yes (Low Risk)	Yes (Low Risk)	Yes (Low Risk)
Lohan et al., 2018	No (High Risk)	Unclear	Unclear	Unclear	Unclear	Unclear	Unclear	Unclear	No (High Risk)	Yes (Low Risk)
Perlee et al., 2019	No (High Risk)	Unclear	Unclear	Yes (Low Risk)	Unclear	Unclear	Yes (Low Risk)	Unclear	Yes (Low Risk)	Yes (Low Risk)
Rogulska et al., 2019	Unclear	Yes (Low Risk)	Unclear	Yes (Low Risk)	Unclear	Unclear	Yes (Low Risk)	Unclear	Yes (Low Risk)	No (High Risk)
Salmenkari et al., 2019	No (High Risk)	Yes (Low Risk)	Unclear	Yes (Low Risk)	Unclear	Unclear	Yes (Low Risk)	Yes (Low Risk)	Yes (Low Risk)	Yes (Low Risk)
Somal et al., 2017	No (High Risk)	Unclear	Unclear	Yes (Low Risk)	Unclear	Unclear	Unclear	Unclear	Yes (Low Risk)	Yes (Low Risk)
Tan et al., 2019	Yes (Low Risk)	Yes (Low Risk)	Yes (Low Risk)	Unclear	Yes (Low Risk)	Unclear	Yes (Low Risk)	Yes (Low Risk)	Yes (Low Risk)	Yes (Low Risk)
Yea et al., 2020	Unclear	Yes (Low Risk)	Unclear	Yes (Low Risk)	Unclear	Unclear	Unclear	Unclear	Yes (Low Risk)	Yes (Low Risk)
Horiuchi et al., 2021	Unclear	Yes (Low Risk)	Unclear	Yes (Low Risk)	Unclear	Unclear	Unclear	Unclear	Yes (Low Risk)	Yes (Low Risk)
Horie et al., 2021	Unclear	Yes (Low Risk)	Yes (Low Risk)	Yes (Low Risk)	Unclear	Unclear	Yes (Low Risk)	Unclear	Yes (Low Risk)	Yes (Low Risk)

### Primary and secondary outcomes

Across the 18 included studies, a total of 325 experiments and 133 distinct outcome measures were reported on our primary and secondary outcomes and are summarized below. Data extraction of outcomes from included studies yielded significant amounts of data given the extensive and varied inflammatory disease models and their specific outcomes. A description of all primary in vivo pre-clinical efficacy and secondary in vitro potency outcomes are reported in Table 4 and 6, respectively. The studies included in our systematic review varied with respect to disease type, MSC source, MSC processing, route of administration, dose, outcome measures, and timing of outcome measurement. Due to this high degree of heterogeneity, meta-analyses were not feasible for the primary and secondary outcome measures. However, similar pre-clinical animal inflammatory models that reported similar outcomes are reported in Table 5 for reference.

**Table 4.** All in vivo outcomes where freshly cultured vs. cryopreserved MSCs have been compared directly are reported.

Study	Animal Model	Outcome	Number (n)	Type and Source of MSCs	Duration of Culture Post-Thaw (hr)	Concentration of MSCs	Pre-Treatment of MSCs	Negative Control (NC)	Positive Control (PC)	p-value for Fresh MSCs vs. control	p-value for Frozen MSCs vs. control	Fresh or Frozen MSC more effective?	p-value for Fresh vs. Frozen comparison
**Acute Lung Injury and Sepsis**													
Devaney et al., 2015	Acute lung injury induced by *E. coli* pneumonia in rats	Arterial oxygenation	10	Human Bone Marrow	0	1×10^7 hMSCs/kg	N/A	N/A	PBS	<0.05	<0.05	↔	NS
		Lung compliance	10	Human Bone Marrow	0	1×10^7 hMSCs/kg	N/A	N/A	PBS	<0.05	<0.05	↔	NS
		BAL protein	10	Human Bone Marrow	0	1×10^7 hMSCs/kg	N/A	N/A	PBS	<0.05	<0.05	↔	NS
		BAL neutrophils	10	Human Bone Marrow	0	1×10^7 hMSCs/kg	N/A	N/A	PBS	<0.05	<0.05	↔	NS
		BAL *E. coli* bacterial load	10	Human Bone Marrow	0	1×10^7 hMSCs/kg	N/A	N/A	PBS	<0.05	<0.05	↔	NS
		BAL IL-6	10	Human Bone Marrow	0	1×10^7 hMSCs/kg	N/A	N/A	PBS	<0.05	<0.05	↔	NS
		BAL IL-10	10	Human Bone Marrow	0	1×10^7 hMSCs/kg	N/A	N/A	PBS	<0.05	<0.05	↔	NS
Cruz et al., 2015	Allergic Airways Inflammation induced by Aspergillus hyphal extract (AHE) exposure in mice.	Large Airway Resistance	10 (Fresh) and 7 (Frozen)	Human Bone Marrow	0	1 × 10^6 viable MSC cells	Frozen MSCs washed 3 times prior to use	Naïve (PBS model)	AHE +PBS, Human Lung Fibroblasts	<0.05	<0.05	↔	NS
		Large Airway Resistance	6	Murine Bone Marrow	0	1 × 10^6 viable MSC cells	Frozen MSCs washed 3 times prior to use	Naïve (PBS model)	AHE +PBS, Human Lung Fibroblasts	<0.05	<0.05	↔	NS
		Overall Tissue Resistance	10 (Fresh) and 7 (Frozen)	Human Bone Marrow	0	1 × 10^6 viable MSC cells	Frozen MSCs washed 3 times prior to use	Naïve (PBS model)	AHE +PBS, Human Lung Fibroblasts	<0.05	<0.05	↔	NS
		Overall Tissue Resistance	6	Murine Bone Marrow	0	1 × 10^6 viable MSC cells	Frozen MSCs washed 3 times prior to use	Naïve (PBS model)	AHE +PBS, Human Lung Fibroblasts	<0.05	<0.05	↔	NS
		Lung Elastance	10 (Fresh) and 7 (Frozen)	Human Bone Marrow	0	1 × 10^6 viable MSC cells	Frozen MSCs washed 3 times prior to use	Naïve (PBS model)	AHE +PBS, Human Lung Fibroblasts	<0.05	<0.05	↔	NS
		Lung Elastance	6	Murine Bone Marrow	0	1 × 10^6 viable MSC cells	Frozen MSCs washed 3 times prior to use	Naïve (PBS model)	AHE +PBS, Human Lung Fibroblasts	<0.05	<0.05	↔	NS
		Inflammation Score	10 (Fresh) and 7 (Frozen)	Human Bone Marrow	0	1 × 10^6 viable MSC cells	Frozen MSCs washed 3 times prior to use	Naïve (PBS model)	AHE +PBS, Human Lung Fibroblasts	<0.05	<0.05	↔	NS
		Inflammation Score	6	Murine Bone Marrow	0	1 × 10^6 viable MSC cells	Frozen MSCs washed 3 times prior to use	Naïve (PBS model)	AHE +PBS, Human Lung Fibroblasts	<0.05	<0.05	↔	NS
		BALF Total Cell Number	10 (Fresh) and 7 (Frozen)	Human Bone Marrow	0	1 × 10^6 viable MSC cells	Frozen MSCs washed 3 times prior to use	Naïve (PBS model)	AHE +PBS, Human Lung Fibroblasts	<0.05	<0.05	↔	NS
		BALF Total Cell Number	6	Murine Bone Marrow	0	1 × 10^6 viable MSC cells	Frozen MSCs washed 3 times prior to use	Naïve (PBS model)	AHE +PBS, Human Lung Fibroblasts	<0.05	<0.05	↔	NS
		BAL Neutrophils	10 (Fresh) and 7 (Frozen)	Human Bone Marrow	0	1 × 10^6 viable MSC cells	Frozen MSCs washed 3 times prior to use	Naïve (PBS model)	AHE +PBS, Human Lung Fibroblasts	<0.05	<0.05	↔	NS
		BAL Neutrophils	6	Murine Bone Marrow	0	1 × 10^6 viable MSC cells	Frozen MSCs washed 3 times prior to use	Naïve (PBS model)	AHE +PBS, Human Lung Fibroblasts	<0.05	<0.05	↔	NS
		BAL Eosinophils	10 (Fresh) and 7 (Frozen)	Human Bone Marrow	0	1 × 10^6 viable MSC cells	Frozen MSCs washed 3 times prior to use	Naïve (PBS model)	AHE +PBS, Human Lung Fibroblasts	<0.05	<0.05	↔	NS
		BAL Eosinophils	6	Murine Bone Marrow	0	1 × 10^6 viable MSC cells	Frozen MSCs washed 3 times prior to use	Naïve (PBS model)	AHE +PBS, Human Lung Fibroblasts	<0.05	<0.05	↔	NS
		BAL Macrophages	10 (Fresh) and 7 (Frozen)	Human Bone Marrow	0	1 × 10^6 viable MSC cells	Frozen MSCs washed 3 times prior to use	Naïve (PBS model)	AHE +PBS, Human Lung Fibroblasts	<0.05	<0.05	↔	NS
		BAL Macrophages	6	Murine Bone Marrow	0	1 × 10^6 viable MSC cells	Frozen MSCs washed 3 times prior to use	Naïve (PBS model)	AHE +PBS, Human Lung Fibroblasts	<0.05	<0.05	↔	NS
		BAL Lymphocytes	10 (Fresh) and 7 (Frozen)	Human Bone Marrow	0	1 × 10^6 viable MSC cells	Frozen MSCs washed 3 times prior to use	Naïve (PBS model)	AHE +PBS, Human Lung Fibroblasts	<0.05	<0.05	↔	NS
		BAL Lymphocytes	6	Murine Bone Marrow	0	1 × 10^6 viable MSC cells	Frozen MSCs washed 3 times prior to use	Naïve (PBS model)	AHE +PBS, Human Lung Fibroblasts	<0.05	<0.05	Frozen better	**<0.05**
		BAL IL-1a	10 (Fresh) and 7 (Frozen)	Human Bone Marrow	0	1 × 10^6 viable MSC cells	Frozen MSCs washed 3 times prior to use	Naïve (PBS model)	AHE +PBS, Human Lung Fibroblasts	<0.05	<0.05	↔	NS
		BAL IL-1a	6	Murine Bone Marrow	0	1 × 10^6 viable MSC cells	Frozen MSCs washed 3 times prior to use	Naïve (PBS model)	AHE +PBS, Human Lung Fibroblasts	<0.05	<0.05	↔	NS
		BAL IL-3	10 (Fresh) and 7 (Frozen)	Human Bone Marrow	0	1 × 10^6 viable MSC cells	Frozen MSCs washed 3 times prior to use	Naïve (PBS model)	AHE +PBS, Human Lung Fibroblasts	<0.05	<0.05	↔	NS
		BAL IL-3	6	Murine Bone Marrow	0	1 × 10^6 viable MSC cells	Frozen MSCs washed 3 times prior to use	Naïve (PBS model)	AHE +PBS, Human Lung Fibroblasts	<0.05	<0.05	↔	NS
		BAL IL-4	10 (Fresh) and 7 (Frozen)	Human Bone Marrow	0	1 × 10^6 viable MSC cells	Frozen MSCs washed 3 times prior to use	Naïve (PBS model)	AHE +PBS, Human Lung Fibroblasts	<0.05	<0.05	↔	NS
		BAL IL-4	6	Murine Bone Marrow	0	1 × 10^6 viable MSC cells	Frozen MSCs washed 3 times prior to use	Naïve (PBS model)	AHE +PBS, Human Lung Fibroblasts	<0.05	<0.05	↔	NS
		BAL IL-5	10 (Fresh) and 7 (Frozen)	Human Bone Marrow	0	1 × 10^6 viable MSC cells	Frozen MSCs washed 3 times prior to use	Naïve (PBS model)	AHE +PBS, Human Lung Fibroblasts	<0.05	<0.05	↔	NS
		BAL IL-5	6	Murine Bone Marrow	0	1 × 10^6 viable MSC cells	Frozen MSCs washed 3 times prior to use	Naïve (PBS model)	AHE +PBS, Human Lung Fibroblasts	<0.05	<0.05	↔	NS
		BAL IL-6	10 (Fresh) and 7 (Frozen)	Human Bone Marrow	0	1 × 10^6 viable MSC cells	Frozen MSCs washed 3 times prior to use	Naïve (PBS model)	AHE +PBS, Human Lung Fibroblasts	<0.05	<0.05	↔	NS
		BAL IL-6	6	Murine Bone Marrow	0	1 × 10^6 viable MSC cells	Frozen MSCs washed 3 times prior to use	Naïve (PBS model)	AHE +PBS, Human Lung Fibroblasts	<0.05	<0.05	↔	NS
		BAL IL-10	10 (Fresh) and 7 (Frozen)	Human Bone Marrow	0	1 × 10^6 viable MSC cells	Frozen MSCs washed 3 times prior to use	Naïve (PBS model)	AHE +PBS, Human Lung Fibroblasts	<0.05	<0.05	↔	NS
		BAL IL-10	6	Murine Bone Marrow	0	1 × 10^6 viable MSC cells	Frozen MSCs washed 3 times prior to use	Naïve (PBS model)	AHE +PBS, Human Lung Fibroblasts	<0.05	<0.05	↔	NS
		BAL IL-12-p40	10 (Fresh) and 7 (Frozen)	Human Bone Marrow	0	1 × 10^6 viable MSC cells	Frozen MSCs washed 3 times prior to use	Naïve (PBS model)	AHE +PBS, Human Lung Fibroblasts	<0.05	<0.05	↔	NS
		BAL IL-12-p40	6	Murine Bone Marrow	0	1 × 10^6 viable MSC cells	Frozen MSCs washed 3 times prior to use	Naïve (PBS model)	AHE +PBS, Human Lung Fibroblasts	<0.05	<0.05	↔	NS
		BAL IL-13	10 (Fresh) and 7 (Frozen)	Human Bone Marrow	0	1 × 10^6 viable MSC cells	Frozen MSCs washed 3 times prior to use	Naïve (PBS model)	AHE +PBS, Human Lung Fibroblasts	<0.05	<0.05	↔	NS
		BAL IL-13	6	Murine Bone Marrow	0	1 × 10^6 viable MSC cells	Frozen MSCs washed 3 times prior to use	Naïve (PBS model)	AHE +PBS, Human Lung Fibroblasts	<0.05	<0.05	↔	NS
		BAL IL-17	10 (Fresh) and 7 (Frozen)	Human Bone Marrow	0	1 × 10^6 viable MSC cells	Frozen MSCs washed 3 times prior to use	Naïve (PBS model)	AHE +PBS, Human Lung Fibroblasts	<0.05	<0.05	Fresh better	**<0.05**
		BAL IL-17	6	Murine Bone Marrow	0	1 × 10^6 viable MSC cells	Frozen MSCs washed 3 times prior to use	Naïve (PBS model)	AHE +PBS, Human Lung Fibroblasts	<0.05	<0.05	↔	NS
		BAL KC	10 (Fresh) and 7 (Frozen)	Human Bone Marrow	0	1 × 10^6 viable MSC cells	Frozen MSCs washed 3 times prior to use	Naïve (PBS model)	AHE +PBS, Human Lung Fibroblasts	<0.05	<0.05	Fresh better	**<0.05**
		BAL KC	6	Murine Bone Marrow	0	1 × 10^6 viable MSC cells	Frozen MSCs washed 3 times prior to use	Naïve (PBS model)	AHE +PBS, Human Lung Fibroblasts	<0.05	<0.05	Frozen better	**<0.05**
		BAL RANTES	10 (Fresh) and 7 (Frozen)	Human Bone Marrow	0	1 × 10^6 viable MSC cells	Frozen MSCs washed 3 times prior to use	Naïve (PBS model)	AHE +PBS, Human Lung Fibroblasts	<0.05	<0.05	↔	NS
		BAL RANTES	6	Murine Bone Marrow	0	1 × 10^6 viable MSC cells	Frozen MSCs washed 3 times prior to use	Naïve (PBS model)	AHE +PBS, Human Lung Fibroblasts	<0.05	<0.05	↔	NS
		IFN-y	10 (Fresh) and 7 (Frozen)	Human Bone Marrow	0	1 × 10^6 viable MSC cells	Frozen MSCs washed 3 times prior to use	Naïve (PBS model)	AHE +PBS, Human Lung Fibroblasts	<0.05	<0.05	↔	NS
		IFN-y	6	Murine Bone Marrow	0	1 × 10^6 viable MSC cells	Frozen MSCs washed 3 times prior to use	Naïve (PBS model)	AHE +PBS, Human Lung Fibroblasts	<0.05	<0.05	↔	NS
Curley et al., 2017	Acute respiratory distress syndrome by intratracheal instillation of *E. coli* in rats.	Arterial Oxygenation (FiO2=0.3)	8–10	Human Umbilical Cord (Frozen) and Bone marrow (Fresh) MSCs	NR	1×10^7 MSCs/kg	N/A	Sham model +PBS	*E. coli*+PBS	<0.05	<0.05	↔	NS
		Arterial Oxygenation (FiO2=1)	8–10	Human Umbilical Cord (Frozen) and Bone marrow (Fresh) MSCs	NR	1×10^7 MSCs/kg	N/A	Sham model +PBS	*E. coli*+PBS	<0.05	<0.05	↔	NS
		Lung Compliance	8–10	Human Umbilical Cord (Frozen) and Bone marrow (Fresh) MSCs	NR	1×10^7 MSCs/kg	N/A	Sham model +PBS	*E. coli*+PBS	<0.05	<0.05	↔	NS
		Wet:Dry Lung Ratio	8–10	Human Umbilical Cord (Frozen) and Bone marrow (Fresh) MSCs	NR	1×10^7 MSCs/kg	N/A	Sham model +PBS	*E. coli*+PBS	<0.05	<0.05	↔	NS
		BAL Neutrophils	8–10	Human Umbilical Cord (Frozen) and Bone marrow (Fresh) MSCs	NR	1×10^7 MSCs/kg	N/A	Sham model +PBS	*E. coli*+PBS	<0.05	<0.05	↔	NS
		BAL Bacteria	8–10	Human Umbilical Cord (Frozen) and Bone marrow (Fresh) MSCs	NR	1×10^7 MSCs/kg	N/A	Sham model +PBS	*E. coli*+PBS	<0.05	<0.05	↔	NS
Bárcia et al., 2017	1) Chronic adjuvant-induced arthritis (AIA) model 2) Hindlimb ischemia model in mice	Arthritis Index	6	Human Umbilical Cord MSCs	0	1.7×10^6 MSCs	Fresh MSCs were cryopreserved and then cultured for up to 5 days	Sham model +PBS	N/A	*P*<0.0001	*P*<0.0001	↔	NS
		Left Paw Volume	6	Human Umbilical Cord MSCs	0	1.7×10^6 MSCs	Fresh MSCs were cryopreserved and then cultured for up to 5 days	Sham model +PBS	N/A	*P*<0.0001	*P*<0.0001	↔	NS
		Right Paw Volume	6	Human Umbilical Cord MSCs	0	1.7×10^6 MSCs	Fresh MSCs were cryopreserved and then cultured for up to 5 days	Sham model +PBS	N/A	*P*<0.0001	*P*<0.0001	↔	NS
		Weight	6	Human Umbilical Cord MSCs	0	1.7×10^6 MSCs	Fresh MSCs were cryopreserved and then cultured for up to 5 days	Sham model +PBS	N/A	*P*<0.0001	*P*<0.0001	↔	NS
		Blood Flow Ratio in Hindlimb D0	12	Human Umbilical Cord MSCs	0	2×10^5 MSCs	Fresh MSCs were cryopreserved and then cultured for up to 5 days	N/A	PBS	NS	NS	↔	NS
		Blood Flow Ratio in Hindlimb D7	12	Human Umbilical Cord MSCs	0	2×10^5 MSCs	Fresh MSCs were cryopreserved and then cultured for up to 5 days	N/A	PBS	*P*=0.008	*P*=0.019	↔	NS
		Blood Flow Ratio in Hindlimb D14	12	Human Umbilical Cord MSCs	0	2×10^5 MSCs	Fresh MSCs were cryopreserved and then cultured for up to 5 days	N/A	PBS	*P*=0.012	*P*=0.031	↔	NS
		Blood Flow Ratio in Hindlimb D21	12	Human Umbilical Cord MSCs	0	2×10^5 MSCs	Fresh MSCs were cryopreserved and then cultured for up to 5 days	N/A	PBS	*P*=0.004	*P*=0.002	↔	NS
Salmenkari et al., 2019	Acute phase and Regenerative Phase of Colitis model in mice	Macroscopic Score	9	Human Bone Marrow	NR	0.5 x 10^6 MSCs	N/A	Sham model with PBS	Colitis +Vehicle	PC: NS	PC: NS	↔	NS
		Colon Weight (% change)	9	Human Bone Marrow	NR	0.5 x 10^6 MSCs	N/A	Sham model with PBS	Colitis +Vehicle	PC: NS NC = *P*=0.001	PC: NS NC: *P*=0.001	↔	NS
		Colon Length	9	Human Bone Marrow	NR	0.5 x 10^6 MSCs	N/A	Sham model with PBS	Colitis +Vehicle	PC: NS NC = *P*=0.018	PC: NS NC: *P*=0.014	↔	NS
		Histopathology Scpre	9	Human Bone Marrow	NR	0.5 x 10^6 MSCs	N/A	Sham model with PBS	Colitis +Vehicle	PC: NS NC = *P*=0.004	PC: NS NC: *P*=0.001	↔	NS
		Regeneration	9	Human Bone Marrow	NR	0.5 x 10^6 MSCs	N/A	Sham model with PBS	Colitis +Vehicle	PC: NS	PC: NS	↔	NS
		IL-1b in colon tissue homogenates	9	Human Bone Marrow	NR	0.5 x 10^6 MSCs	N/A	Sham model with PBS	Colitis +Vehicle	PC: NS	PC: NS	↔	NS
		TNFa in colon tissue homogenates	9	Human Bone Marrow	NR	0.5 x 10^6 MSCs	N/A	Sham model with PBS	Colitis +Vehicle	PC: NS	PC: NS	↔	NS
		IL-1b mRNA in colon	9	Human Bone Marrow	NR	0.5 x 10^6 MSCs	N/A	Sham model with PBS	Colitis +Vehicle	PC: NS	PC: NS	↔	NS
		Corticosterone in colon tissue homogenates	9	Human Bone Marrow	NR	0.5 x 10^6 MSCs	N/A	Sham model with PBS	Colitis +Vehicle	PC: NS	PC: NS	↔	NS
		Tissue ACE levels	9	Human Bone Marrow	NR	0.5 x 10^6 MSCs	N/A	Sham model with PBS	Colitis +Vehicle	PC: NS	PC: *P*<0.05	↔	NS
		Atgr1a mRNA expression	9	Human Bone Marrow	NR	0.5 x 10^6 MSCs	N/A	Sham model with PBS	Colitis +Vehicle	PC: NS	PC: NS	↔	NS
		ACE shedding	9	Human Bone Marrow	NR	0.5 x 10^6 MSCs	N/A	Sham model with PBS	Colitis +Vehicle	PC: NS	PC: *P*<0.001	↔	NS
Somal et al., 2017	Wound Healing of surgical dorsal limb wound in rats	Wound Area D0	3	Caprine Amniotic Fluid	NR	1 × 10^6 MSC cells	N/A	N/A	PBS	NS	NS	↔	NS
		Wound Area D7	3	Caprine Amniotic Fluid	NR	1 × 10^6 MSC cells	N/A	N/A	PBS	*P*<0.05	*P*<0.05	↔	NS
		Wound Area D14	3	Caprine Amniotic Fluid	NR	1 × 10^6 MSC cells	N/A	N/A	PBS	NS	NS	↔	NS
		Wound Area D21	3	Caprine Amniotic Fluid	NR	1 × 10^6 MSC cells	N/A	N/A	PBS	NS	NS	↔	NS
		Wound Area D28	3	Caprine Amniotic Fluid	NR	1 × 10^6 MSC cells	N/A	N/A	PBS	NS	NS	↔	NS
		% Wound Contraction D7	3	Caprine Amniotic Fluid	NR	1 × 10^6 MSC cells	N/A	N/A	PBS	*P*<0.05	NS	↔	NS
		% Wound Contraction D14	3	Caprine Amniotic Fluid	NR	1 × 10^6 MSC cells	N/A	N/A	PBS	NS	NS	↔	NS
		% Wound Contraction D21	3	Caprine Amniotic Fluid	NR	1 × 10^6 MSC cells	N/A	N/A	PBS	NS	NS	↔	NS
		% Wound Contraction D28	3	Caprine Amniotic Fluid	NR	1 × 10^6 MSC cells	N/A	N/A	PBS	NS	NS	↔	NS
		Epithelization	3	Caprine Amniotic Fluid	NR	1 × 10^6 MSC cells	N/A	N/A	PBS	*P*<0.05	*P*<0.05	↔	NS
		Neovascularization	3	Caprine Amniotic Fluid	NR	1 × 10^6 MSC cells	N/A	N/A	PBS	*P*<0.05	*P*<0.05	↔	NS
		Collagen Thickness	3	Caprine Amniotic Fluid	NR	1 × 10^6 MSC cells	N/A	N/A	PBS	*P*<0.05	*P*<0.05	↔	NS
		Collagen Density	3	Caprine Amniotic Fluid	NR	1 × 10^6 MSC cells	N/A	N/A	PBS	*P*<0.05	*P*<0.05	↔	NS
		Wound Area D0	3	Caprine Amniotic Sac	NR	1 × 10^6 MSC cells	N/A	N/A	PBS	NS	NS	↔	NS
		Wound Area D7	3	Caprine Amniotic Sac	NR	1 × 10^6 MSC cells	N/A	N/A	PBS	NS	NS	↔	NS
		Wound Area D14	3	Caprine Amniotic Sac	NR	1 × 10^6 MSC cells	N/A	N/A	PBS	NS	NS	↔	NS
		Wound Area D21	3	Caprine Amniotic Sac	NR	1 × 10^6 MSC cells	N/A	N/A	PBS	NS	NS	↔	NS
		Wound Area D28	3	Caprine Amniotic Sac	NR	1 × 10^6 MSC cells	N/A	N/A	PBS	NS	NS	↔	NS
		% Wound Contraction D7	3	Caprine Amniotic Sac	NR	1 × 10^6 MSC cells	N/A	N/A	PBS	NS	NS	↔	NS
		% Wound Contraction D14	3	Caprine Amniotic Sac	NR	1 × 10^6 MSC cells	N/A	N/A	PBS	NS	NS	↔	NS
		% Wound Contraction D21	3	Caprine Amniotic Sac	NR	1 × 10^6 MSC cells	N/A	N/A	PBS	NS	NS	↔	NS
		% Wound Contraction D28	3	Caprine Amniotic Sac	NR	1 × 10^6 MSC cells	N/A	N/A	PBS	NS	NS	↔	NS
		Epithelization	3	Caprine Amniotic Sac	NR	1 × 10^6 MSC cells	N/A	N/A	PBS	*P*<0.05	*P*<0.05	↔	NS
		Neovascularization	3	Caprine Amniotic Sac	NR	1 × 10^6 MSC cells	N/A	N/A	PBS	*P*<0.05	*P*<0.05	↔	NS
		Collagen Thickness	3	Caprine Amniotic Sac	NR	1 × 10^6 MSC cells	N/A	N/A	PBS	NS	*P*<0.05	↔	NS
		Collagen Density	3	Caprine Amniotic Sac	NR	1 × 10^6 MSC cells	N/A	N/A	PBS	*P*<0.05	*P*<0.05	↔	NS
		Wound Area D0	3	Caprine Wharton’s Jelly	NR	1 × 10^6 MSC cells	N/A	N/A	PBS	NS	NS	↔	NS
		Wound Area D7	3	Caprine Wharton’s Jelly	NR	1 × 10^6 MSC cells	N/A	N/A	PBS	*P*<0.05	NS	↔	NS
		Wound Area D14	3	Caprine Wharton’s Jelly	NR	1 × 10^6 MSC cells	N/A	N/A	PBS	NS	NS	↔	NS
		Wound Area D21	3	Caprine Wharton’s Jelly	NR	1 × 10^6 MSC cells	N/A	N/A	PBS	NS	NS	↔	NS
		Wound Area D28	3	Caprine Wharton’s Jelly	NR	1 × 10^6 MSC cells	N/A	N/A	PBS	NS	NS	↔	NS
		% Wound Contraction D7	3	Caprine Wharton’s Jelly	NR	1 × 10^6 MSC cells	N/A	N/A	PBS	*P*<0.05	NS	↔	NS
		% Wound Contraction D14	3	Caprine Wharton’s Jelly	NR	1 × 10^6 MSC cells	N/A	N/A	PBS	NS	NS	↔	NS
		% Wound Contraction D21	3	Caprine Wharton’s Jelly	NR	1 × 10^6 MSC cells	N/A	N/A	PBS	NS	NS	↔	NS
		% Wound Contraction D28	3	Caprine Wharton’s Jelly	NR	1 × 10^6 MSC cells	N/A	N/A	PBS	NS	NS	↔	NS
		Epithelization	3	Caprine Wharton’s Jelly	NR	1 × 10^6 MSC cells	N/A	N/A	PBS	*P*<0.05	*P*<0.05	↔	NS
		Neovascularization	3	Caprine Wharton’s Jelly	NR	1 × 10^6 MSC cells	N/A	N/A	PBS	*P*<0.05	*P*<0.05	↔	NS
		Collagen Thickness	3	Caprine Wharton’s Jelly	NR	1 × 10^6 MSC cells	N/A	N/A	PBS	*P*<0.05	*P*<0.05	↔	NS
		Collagen Density	3	Caprine Wharton’s Jelly	NR	1 × 10^6 MSC cells	N/A	N/A	PBS	*P*<0.05	*P*<0.05	↔	NS
		Wound Area D0	3	Caprine Cord Blood	NR	1 × 10^6 MSC cells	N/A	N/A	PBS	NS	NS	↔	NS
		Wound Area D7	3	Caprine Cord Blood	NR	1 × 10^6 MSC cells	N/A	N/A	PBS	*P*<0.05	NS	↔	NS
		Wound Area D14	3	Caprine Cord Blood	NR	1 × 10^6 MSC cells	N/A	N/A	PBS	NS	NS	↔	NS
		Wound Area D21	3	Caprine Cord Blood	NR	1 × 10^6 MSC cells	N/A	N/A	PBS	NS	NS	↔	NS
		Wound Area D28	3	Caprine Cord Blood	NR	1 × 10^6 MSC cells	N/A	N/A	PBS	NS	NS	↔	NS
		% Wound Contraction D7	3	Caprine Cord Blood	NR	1 × 10^6 MSC cells	N/A	N/A	PBS	*P*<0.05	NS	↔	NS
		% Wound Contraction D14	3	Caprine Cord Blood	NR	1 × 10^6 MSC cells	N/A	N/A	PBS	NS	NS	↔	NS
		% Wound Contraction D21	3	Caprine Cord Blood	NR	1 × 10^6 MSC cells	N/A	N/A	PBS	NS	NS	↔	NS
		% Wound Contraction D28	3	Caprine Cord Blood	NR	1 × 10^6 MSC cells	N/A	N/A	PBS	NS	NS	↔	NS
		Epithelization	3	Caprine Cord Blood	NR	1 × 10^6 MSC cells	N/A	N/A	PBS	*P*<0.05	*P*<0.05	↔	NS
		Neovascularization	3	Caprine Cord Blood	NR	1 × 10^6 MSC cells	N/A	N/A	PBS	*P*<0.05	*P*<0.05	↔	NS
		Collagen Thickness	3	Caprine Cord Blood	NR	1 × 10^6 MSC cells	N/A	N/A	PBS	NS	NS	↔	NS
		Collagen Density	3	Caprine Cord Blood	NR	1 × 10^6 MSC cells	N/A	N/A	PBS	*P*<0.05	NS	Frozen better	***P*<0.05**
Lohan et al., 2018	Corneal Transplantation in rats	Opacity Score, measured from day 5 post-implantation to day 30	Fresh = 13, Frozen = 10	Rat Bone Marrow	0	1×10^6 MSC	Frozen MSCs pre-treated with allogenic splenocytes, and co-intervention with MMF. No MMF for Fresh MSCs.	N/A	Transplantation +No treatment	NS	NS	↔	NR
		Neovascularization Score, measured from day 5 post-implantation to day 30	Fresh = 13, Frozen = 10	Rat Bone Marrow	0	1×10^6 MSC	Frozen MSCs pre-treated with allogenic splenocytes, and co-intervention with MMF. No MMF for Fresh MSCs.	N/A	Transplantation +No treatment	*P*<0.001	NS	↔	NR
Gramlich et al., 2016	Retinal ischemia/reperfusion model in mice	Retinal ganglion cells/mm^2	Fresh = 10, Frozen = 8	Human MSCs	<1 hr	3×10^4 MSC	N/A	Sham model	PBS	*P*=0.019	*P*=0.024	↔	NS
Perlee et al., 2019	Pneumosepsis Caused by Klebsiella pneumoniae	Lung Bacterial Load at 16 hours	8	Human Adipose Tissue	0	1×10^6 ASCs	MSCs infused at 1 or 6 hours after infection.	N/A	PBS	NS	*P*<0.001	↔	NS
		Lung Bacterial Load at 48 hours	8	Human Adipose Tissue	0	1×10^6 ASCs	MSCs infused at 1 or 6 hours after infection.	N/A	PBS	*P*<0.0001	*P*<0.001	↔	NS
		Blood Bacterial Load at 16 hours	8	Human Adipose Tissue	0	1×10^6 ASCs	MSCs infused at 1 or 6 hours after infection.	N/A	PBS	NS	NS	↔	NS
		Blood Bacterial Load at 48 hours	8	Human Adipose Tissue	0	1×10^6 ASCs	MSCs infused at 1 or 6 hours after infection.	N/A	PBS	*P*<0.001	*P*<0.001	↔	NS
		Liver Bacterial Load at 16 hours	8	Human Adipose Tissue	0	1×10^6 ASCs	MSCs infused at 1 or 6 hours after infection.	N/A	PBS	NS	NS	↔	NS
		Liver Bacterial Load at 48 hours	8	Human Adipose Tissue	0	1×10^6 ASCs	MSCs infused at 1 or 6 hours after infection.	N/A	PBS	*P*<0.0001	*P*<0.001	↔	NS
		Spleen Bacterial Load at 16 hours	8	Human Adipose Tissue	0	1×10^6 ASCs	MSCs infused at 1 or 6 hours after infection.	N/A	PBS	NS	NS	↔	NS
		Spleen Bacterial Load at 48 hours	8	Human Adipose Tissue	0	1×10^6 ASCs	MSCs infused at 1 or 6 hours after infection.	N/A	PBS	*P*<0.001	*P*<0.01	↔	NS
		Lung TNFa at 16 hours	8	Human Adipose Tissue	0	1×10^6 ASCs	MSCs infused at 1 or 6 hours after infection.	N/A	PBS	*P*<0.0001	*P*<0.05	↔	NS
		Lung TNFa at 48 hours	8	Human Adipose Tissue	0	1×10^6 ASCs	MSCs infused at 1 or 6 hours after infection.	N/A	PBS	*P*<0.001	*P*<0.05	↔	NS
		Lung IL-1b at 16 hours	8	Human Adipose Tissue	0	1×10^6 ASCs	MSCs infused at 1 or 6 hours after infection.	N/A	PBS	*P*<0.05	*P*<0.01	↔	NS
		Lung IL-1b at 48 hours	8	Human Adipose Tissue	0	1×10^6 ASCs	MSCs infused at 1 or 6 hours after infection.	N/A	PBS	*P*<0.001	*P*<0.05	↔	NS
		Lung IL-6 at 16 hours	8	Human Adipose Tissue	0	1×10^6 ASCs	MSCs infused at 1 or 6 hours after infection.	N/A	PBS	*P*<0.05	*P*<0.01	↔	NS
		Lung IL-6 at 48 hours	8	Human Adipose Tissue	0	1×10^6 ASCs	MSCs infused at 1 or 6 hours after infection.	N/A	PBS	*P*<0.01	NS	↔	NS
		MIP-2 at 16 hours	8	Human Adipose Tissue	0	1×10^6 ASCs	MSCs infused at 1 or 6 hours after infection.	N/A	PBS	*P*<0.05	*P*<0.01	↔	NS
		MIP-2 at 48 hours	8	Human Adipose Tissue	0	1×10^6 ASCs	MSCs infused at 1 or 6 hours after infection.	N/A	PBS	*P*<0.001	*P*<0.05	↔	NS
Horie et al., 2020a	*E. coli*-induced lung injury.	Arterial Oxygenation	8	Human Umbilical Cord	0	1 × 10^7 MSCs/kg	Isolated CD362+MSCs for use	N/A	PBS	*P*<0.05	*P*<0.05	↔	NS
		Lung Wet:Dry Ratio	8	Human Umbilical Cord	0	1 × 10^7 MSCs/kg	Isolated CD362+MSCs for use	N/A	PBS	NS	NS	↔	NS
		Lung Compliance	8	Human Umbilical Cord	0	1 × 10^7 MSCs/kg	Isolated CD362+MSCs for use	N/A	PBS	*P*<0.05	NS	↔	NS
		BAL *E. coli* Counts	8	Human Umbilical Cord	0	1 × 10^7 MSCs/kg	Isolated CD362+MSCs for use	N/A	PBS	*P*<0.05	*P*<0.05	↔	NS
		BAL WCC levels	8	Human Umbilical Cord	0	1 × 10^7 MSCs/kg	Isolated CD362+MSCs for use	N/A	PBS	*P*<0.05	*P*<0.05	↔	NS
		BAL Neutrophils	8	Human Umbilical Cord	0	1 × 10^7 MSCs/kg	Isolated CD362+MSCs for use	N/A	PBS	*P*<0.05	*P*<0.05	↔	NS
		BAL IL-1b	8	Human Umbilical Cord	0	1 × 10^7 MSCs/kg	Isolated CD362+MSCs for use	N/A	PBS	*P*<0.05	*P*<0.05	↔	NS
		BAL CINC-1	8	Human Umbilical Cord	0	1 × 10^7 MSCs/kg	Isolated CD362+MSCs for use	N/A	PBS	NS	NS	↔	NS
		BAL IL-6	8	Human Umbilical Cord	0	1 × 10^7 MSCs/kg	Isolated CD362+MSCs for use	N/A	PBS	*P*<0.05	*P*<0.05	↔	NS
Horie et al., 2020a	Ventilator-induced Lung Injury	Arterial Oxygenation	Fresh, n=7–8; Cryopreserved, n=5– 6	Human Bone Marrow	NR	1×10^7 MSCs/ kg	Pre-activated MSCs (fresh and frozen were also used)	Sham model	PBS	*P*<0.001	*P*<0.001	↔	NS
		Lung Compliance	Fresh, n=7–8; Cryopreserved, n=5– 6	Human Bone Marrow	NR	1×10^7 MSCs/ kg	Pre-activated MSCs (fresh and frozen were also used)	Sham model	PBS	NS	NS	↔	NS
		Lung Wet:Dry Ratio	Fresh, n=7–8; Cryopreserved, n=5– 6	Human Bone Marrow	NR	1×10^7 MSCs/ kg	Pre-activated MSCs (fresh and frozen were also used)	Sham model	PBS	*P*<0.05	*P*<0.05	↔	NS
		BAL Protein	Fresh, n=7–8; Cryopreserved, n=5– 6	Human Bone Marrow	NR	1×10^7 MSCs/ kg	Pre-activated MSCs (fresh and frozen were also used)	Sham model	PBS	NS	NS	↔	NS
		Percentage of Alveolar Airspace	Fresh, n=8; Cryopreserved, n=6	Human Bone Marrow	NR	1×10^7 MSCs/ kg	Pre-activated MSCs (fresh and frozen were also used)	Sham model	PBS	*P*<0.001	*P*<0.001	↔	NS
		BAL Neutrophils	Fresh, n=6–8; Cryopreserved, n=5–6	Human Bone Marrow	NR	1×10^7 MSCs/ kg	Pre-activated MSCs (fresh and frozen were also used)	Sham model	PBS	*P*<0.05	*P*<0.01	↔	NS
		BAL CINC-1	Fresh, n=6–8; Cryopreserved, n=5–6	Human Bone Marrow	NR	1×10^7 MSCs/ kg	Pre-activated MSCs (fresh and frozen were also used)	Sham model	PBS	*P*<0.05	*P*<0.05	↔	NS
		BAL IL-6	Fresh, n=6–8; Cryopreserved, n=5–6	Human Bone Marrow	NR	1×10^7 MSCs/ kg	Pre-activated MSCs (fresh and frozen were also used)	Sham model	PBS	*P*<0.05	*P*<0.001	↔	NS
		BAL IL-10	Fresh, n=6–8; Cryopreserved, n=5–6	Human Bone Marrow	NR	1×10^7 MSCs/ kg	Pre-activated MSCs (fresh and frozen were also used)	Sham model	PBS	NS	NS	↔	NS
		BAL KGF	Fresh, n=6–8; Cryopreserved, n=5–6	Human Bone Marrow	NR	1×10^7 MSCs/ kg	Pre-activated MSCs (fresh and frozen were also used)	Sham model	PBS	NS	NS	↔	NS
		BAL PGE2	Fresh, n=6–8; Cryopreserved, n=5–6	Human Bone Marrow	NR	1×10^7 MSCs/ kg	Pre-activated MSCs (fresh and frozen were also used)	Sham model	PBS	NS	NS	↔	NS
Tan et al., 2019	Polymicrobial sepsis induced by cecal-ligation-and-puncture (CLP)	%CD11b+/*E. coli*+cells in Peritoneal Fluid	Fresh, n=12; Cryopreserved, n=11	Human Bone Marrow	0	2.5×10^5 MSC cells	N/A	Sham model	PBS	*P*<0.0001	*P*<0.0001	↔	NS
		Peritoneal CFU #	Fresh, n=12; Cryopreserved, n=11	Human Bone Marrow	0	2.5×10^5 MSC cells	N/A	Sham model	PBS	NS	NS	↔	NS
		Plasma Lactate	Fresh, n=12; Cryopreserved, n=11	Human Bone Marrow	0	2.5×10^5 MSC cells	N/A	Sham model	PBS	*P*<0.05	*P*<0.05	↔	NS
		Plasma CCL5	Fresh, n=12; Cryopreserved, n=11	Human Bone Marrow	0	2.5×10^5 MSC cells	N/A	Sham model	PBS	NS	*P*<0.01	↔	NS
		Plasma JE	Fresh, n=12; Cryopreserved, n=11	Human Bone Marrow	0	2.5×10^5 MSC cells	N/A	Sham model	PBS	NS	NS	↔	NS
		Plasma KC	Fresh, n=12; Cryopreserved, n=11	Human Bone Marrow	0	2.5×10^5 MSC cells	N/A	Sham model	PBS	*P*<0.05	NS	↔	NS
		Plasma LIX	Fresh, n=12; Cryopreserved, n=11	Human Bone Marrow	0	2.5×10^5 MSC cells	N/A	Sham model	PBS	NS	NS	↔	NS
		Plasma IL-10	Fresh, n=12; Cryopreserved, n=11	Human Bone Marrow	0	2.5×10^5 MSC cells	N/A	Sham model	PBS	NS	NS	↔	NS
		Plasma IL-1b	Fresh, n=12; Cryopreserved, n=11	Human Bone Marrow	0	2.5×10^5 MSC cells	N/A	Sham model	PBS	NS	NS	↔	NS
Bharti et al., 2020	Wound healing model with 2×2 cm^2 full-thickness excision skin wound in guinea pigs	Percent wound contraction D7	5	Dog Bone Marrow	NR	1×10^6 MSC cells	MSCs attached to polypropylene mesh of 2×2 cm2 size	N/A	Antibiotic only, Mesh only, and MSCs only as control groups	NS	NS	↔	NS
		Percent wound contraction D14	5	Dog Bone Marrow	NR	1×10^6 MSC cells	MSCs attached to polypropylene mesh of 2×2 cm2 size	N/A	Antibiotic only, Mesh only, and MSCs only as control groups	*P*<0.05	*P*<0.05	↔	NS
		Percent wound contraction D21	5	Dog Bone Marrow	NR	1×10^6 MSC cells	MSCs attached to polypropylene mesh of 2×2 cm2 size	N/A	Antibiotic only, Mesh only, and MSCs only as control groups	*P*<0.05	*P*<0.05	↔	NS
		Percent wound contraction D28	5	Dog Bone Marrow	NR	1×10^6 MSC cells	MSCs attached to polypropylene mesh of 2×2 cm2 size	N/A	Antibiotic only, Mesh only, and MSCs only as control groups	*P*<0.05	*P*<0.05	↔	NS
		Epithelialization	5	Dog Bone Marrow	NR	1×10^6 MSC cells	MSCs attached to polypropylene mesh of 2×2 cm2 size	N/A	Antibiotic only, Mesh only, and MSCs only as control groups	*P*<0.05	*P*<0.05	↔	NS
		Neovascularization	5	Dog Bone Marrow	NR	1×10^6 MSC cells	MSCs attached to polypropylene mesh of 2×2 cm2 size	N/A	Antibiotic only, Mesh only, and MSCs only as control groups	*P*<0.05	*P*<0.05	↔	NS
		Collagen Density	5	Dog Bone Marrow	NR	1×10^6 MSC cells	MSCs attached to polypropylene mesh of 2×2 cm2 size	N/A	Antibiotic only, Mesh only, and MSCs only as control groups	*P*<0.05	*P*<0.05	↔	NS
		Collagen Thickness	5	Dog Bone Marrow	NR	1×10^6 MSC cells	MSCs attached to polypropylene mesh of 2×2 cm2 size	N/A	Antibiotic only, Mesh only, and MSCs only as control groups	*P*<0.05	*P*<0.05	↔	NS
Rogulska et al., 2019	Wound Healing of Full-thickness excisional skin wounds in mice	Percent Wound Closure D3	14	Human Adipose Tissue	24 hours	0.25‐0.3×10^6 cells in 50 μl	MSCs placed on 3D gel containing PPP, 0.2 M sucrose, 1% DMSO	N/A	Spontaneous healing, and 3D gel containing PPP, 0.2 M sucrose, 1% DMSO alone	*P*<0.05	*P*<0.05	↔	NS
		Percent Wound Closure D7	14	Human Adipose Tissue	24 hours	0.25‐0.3×10^6 cells in 50 μl	MSCs placed on 3D gel containing PPP, 0.2 M sucrose, 1% DMSO	N/A	Spontaneous healing, and 3D gel containing PPP, 0.2 M sucrose, 1% DMSO alone	*P*<0.05	*P*<0.05	↔	NS
		Percent Wound Closure D14	14	Human Adipose Tissue	24 hours	0.25‐0.3×10^6 cells in 50 μl	MSCs placed on 3D gel containing PPP, 0.2 M sucrose, 1% DMSO	N/A	Spontaneous healing, and 3D gel containing PPP, 0.2 M sucrose, 1% DMSO alone	*P*<0.05	*P*<0.05	↔	NS
		Percent Wound Closure D28	14	Human Adipose Tissue	24 hours	0.25‐0.3×10^6 cells in 50 μl	MSCs placed on 3D gel containing PPP, 0.2 M sucrose, 1% DMSO	N/A	Spontaneous healing, and 3D gel containing PPP, 0.2 M sucrose, 1% DMSO alone	*P*<0.05	*P*<0.05	↔	NS
Khan et al., 2019	Acute Spinal Cord Injury in dogs	Motor activity of hind limbs assessed by using the canine Basso Beattie Bresnahan (cBBB) score at Week 1	4	Dog Adipose Tissue	0	1×10^7 MSC cells	Lentivirus Mediated HO-1 Gene Insertion into Ad- MSCs.	N/A	Fresh MSCs expressing GFP only.	NS	NS	↔	NS
		cBBB score at Week 2	4	Dog Adipose Tissue	0	1×10^7 MSC cells	Lentivirus Mediated HO-1 Gene Insertion into Ad- MSCs.	N/A	Fresh MSCs expressing GFP only.	NS	NS	↔	NS
		cBBB score at Week 3	4	Dog Adipose Tissue	0	1×10^7 MSC cells	Lentivirus Mediated HO-1 Gene Insertion into Ad- MSCs.	N/A	Fresh MSCs expressing GFP only.	NS	NS	↔	NS
		cBBB score at Week 4	4	Dog Adipose Tissue	0	1×10^7 MSC cells	Lentivirus Mediated HO-1 Gene Insertion into Ad- MSCs.	N/A	Fresh MSCs expressing GFP only.	*P*<0.05	NS	↔	NS
		% age of gross lesion area	4	Dog Adipose Tissue	0	1×10^7 MSC cells	Lentivirus Mediated HO-1 Gene Insertion into Ad- MSCs.	N/A	Fresh MSCs expressing GFP only.	NS	NS	↔	NS
		Fibrotic areas relative to normal	4	Dog Adipose Tissue	0	1×10^7 MSC cells	Lentivirus Mediated HO-1 Gene Insertion into Ad- MSCs.	Normal (no SCI)	Fresh MSCs expressing GFP only.	*P*<0.05	NS	↔	NS
		Myelinated areas relative to normal	4	Dog Adipose Tissue	0	1×10^7 MSC cells	Lentivirus Mediated HO-1 Gene Insertion into Ad- MSCs.	Normal (no SCI)	Fresh MSCs expressing GFP only.	*P*<0.05	NS	↔	NS
Yea et al., 2020	Wound healing in rats	Total macroscopic score at 2 weeks	4	Human Umbilical Cord	NR	1×10^6 MSC cells	N/A	Cryoprotectant and PBS	Fresh-MSCs	*P*=0.001	*P*=0.04	↔	NS
		Total macroscopic score at 4 weeks	4	Human Umbilical Cord	NR	1×10^6 MSC cells	N/A	Cryoprotectant and PBS	Fresh-MSCs	*P*=0.001	*P*<0.05	↔	NS
		Total degeneration score at 2 weeks	4	Human Umbilical Cord	NR	1×10^6 MSC cells	N/A	Cryoprotectant and PBS	Fresh-MSCs	*P*<0.001	*P*<0.001	↔	NS
		Total degeneration score at 4 weeks	4	Human Umbilical Cord	NR	1×10^6 MSC cells	N/A	Cryoprotectant and PBS	Fresh-MSCs	*P*<0.05	*P*<0.05	↔	NS
		Fibre structure at 2 weeks	4	Human Umbilical Cord	NR	1×10^6 MSC cells	N/A	Cryoprotectant and PBS	Fresh-MSCs	NS	NS	↔	NS
		Fibre structure at 4 weeks	4	Human Umbilical Cord	NR	1×10^6 MSC cells	N/A	Cryoprotectant and PBS	Fresh-MSCs	*P*<0.05	*P*<0.05	↔	NS
		Fibre arrangement at 2 weeks	4	Human Umbilical Cord	NR	1×10^6 MSC cells	N/A	Cryoprotectant and PBS	Fresh-MSCs	NS	NS	↔	NS
		Fibre arrangement at 4 weeks	4	Human Umbilical Cord	NR	1×10^6 MSC cells	N/A	Cryoprotectant and PBS	Fresh-MSCs	*P*<0.05	*P*<0.05	↔	NS
		Rounding of nuclei at 2 weeks	4	Human Umbilical Cord	NR	1×10^6 MSC cells	N/A	Cryoprotectant and PBS	Fresh-MSCs	NS	NS	↔	NS
		Rounding of nuclei at 4 weeks	4	Human Umbilical Cord	NR	1×10^6 MSC cells	N/A	Cryoprotectant and PBS	Fresh-MSCs	*P*<0.05	*P*<0.05	↔	NS
		Variations in cellularity at 2 weeks	4	Human Umbilical Cord	NR	1×10^6 MSC cells	N/A	Cryoprotectant and PBS	Fresh-MSCs	NS	NS	↔	NS
		Variations in cellularity at 4 weeks	4	Human Umbilical Cord	NR	1×10^6 MSC cells	N/A	Cryoprotectant and PBS	Fresh-MSCs	*P*<0.05	*P*<0.05	↔	NS
		Decreased stainability at 2 weeks	4	Human Umbilical Cord	NR	1×10^6 MSC cells	N/A	Cryoprotectant and PBS	Fresh-MSCs	NS	NS	↔	NS
		Decreased stainability at 4 weeks	4	Human Umbilical Cord	NR	1×10^6 MSC cells	N/A	Cryoprotectant and PBS	Fresh-MSCs	*P*<0.05	*P*<0.05	↔	NS
		Hyalinization at 2 weeks	4	Human Umbilical Cord	NR	1×10^6 MSC cells	N/A	Cryoprotectant and PBS	Fresh-MSCs	NS	NS	↔	NS
		Hyalinization at 4 weeks	4	Human Umbilical Cord	NR	1×10^6 MSC cells	N/A	Cryoprotectant and PBS	Fresh-MSCs	*P*<0.05	*P*<0.05	↔	NS
		Inflammation at 2 weeks	4	Human Umbilical Cord	NR	1×10^6 MSC cells	N/A	Cryoprotectant and PBS	Fresh-MSCs	NS	NS	↔	NS
		Inflammation at 4 weeks	4	Human Umbilical Cord	NR	1×10^6 MSC cells	N/A	Cryoprotectant and PBS	Fresh-MSCs	*P*<0.05	*P*<0.05	↔	NS
		Fibroblast density at 2 weeks	4	Human Umbilical Cord	NR	1×10^6 MSC cells	N/A	Cryoprotectant and PBS	Fresh-MSCs	NS	NS	↔	NS
		Fibroblast density at 4 weeks	4	Human Umbilical Cord	NR	1×10^6 MSC cells	N/A	Cryoprotectant and PBS	Fresh-MSCs	*P*<0.05	*P*<0.05	↔	NS
		Nuclear aspect ratio at 2 weeks	4	Human Umbilical Cord	NR	1×10^6 MSC cells	N/A	Cryoprotectant and PBS	Fresh-MSCs	NS	NS	↔	NS
		Nuclear aspect ration at 4 weeks	4	Human Umbilical Cord	NR	1×10^6 MSC cells	N/A	Cryoprotectant and PBS	Fresh-MSCs	*P*<0.05	*P*<0.05	↔	NS
		Nuclear orientation at 2 weeks	4	Human Umbilical Cord	NR	1×10^6 MSC cells	N/A	Cryoprotectant and PBS	Fresh-MSCs	*P*<0.05	*P*<0.05	↔	NS
		Nuclear orientation at 4 weeks	4	Human Umbilical Cord	NR	1×10^6 MSC cells	N/A	Cryoprotectant and PBS	Fresh-MSCs	*P*<0.05	*P*<0.05	↔	NS
		Collagen organization at 2 weeks	4	Human Umbilical Cord	NR	1×10^6 MSC cells	N/A	Cryoprotectant and PBS	Fresh-MSCs	*P*<0.05	*P*<0.05	↔	NS
		Collagen organization at 4 weeks	4	Human Umbilical Cord	NR	1×10^6 MSC cells	N/A	Cryoprotectant and PBS	Fresh-MSCs	*P*<0.05	*P*<0.05	↔	NS
		Collagen fibre coherence at 2 weeks	4	Human Umbilical Cord	NR	1×10^6 MSC cells	N/A	Cryoprotectant and PBS	Fresh-MSCs	NS	NS	↔	NS
		Collagen fibre coherence at 4 weeks	4	Human Umbilical Cord	NR	1×10^6 MSC cells	N/A	Cryoprotectant and PBS	Fresh-MSCs	*P*<0.05	*P*<0.05	↔	NS
		GAG-rich area at 2 weeks	4	Human Umbilical Cord	NR	1×10^6 MSC cells	N/A	Cryoprotectant and PBS	Fresh-MSCs	*P*<0.05	*P*<0.05	↔	NS
		GAG-rich area at 4 weeks	4	Human Umbilical Cord	NR	1×10^6 MSC cells	N/A	Cryoprotectant and PBS	Fresh-MSCs	*P*<0.05	*P*<0.05	↔	NS
		Ultimate failure load at 2 weeks	8	Human Umbilical Cord	NR	1×10^6 MSC cells	N/A	Cryoprotectant and PBS	Fresh-MSCs	*P*<0.05	*P*<0.05	↔	NS
		Ultimate failure load at 4 weeks	8	Human Umbilical Cord	NR	1×10^6 MSC cells	N/A	Cryoprotectant and PBS	Fresh-MSCs	*P*<0.05	*P*<0.05	↔	NS
		Tendon stiffness at 2 weeks	8	Human Umbilical Cord	NR	1×10^6 MSC cells	N/A	Cryoprotectant and PBS	Fresh-MSCs	*P*<0.05	*P*<0.05	↔	NS
		Tendon stiffness at 4 weeks	8	Human Umbilical Cord	NR	1×10^6 MSC cells	N/A	Cryoprotectant and PBS	Fresh-MSCs	NS	NS	↔	NS
		Ultimate stress at 2 weeks	8	Human Umbilical Cord	NR	1×10^6 MSC cells	N/A	Cryoprotectant and PBS	Fresh-MSCs	*P*<0.05	*P*<0.05	↔	NS
		Ultimate stress at 4 weeks	8	Human Umbilical Cord	NR	1×10^6 MSC cells	N/A	Cryoprotectant and PBS	Fresh-MSCs	*P*<0.05	*P*<0.05	↔	NS
		Cross-sectional area at 2 weeks	8	Human Umbilical Cord	NR	1×10^6 MSC cells	N/A	Cryoprotectant and PBS	Fresh-MSCs	*P*<0.05	*P*<0.05	↔	NS
		Cross-sectional area at 4 weeks	8	Human Umbilical Cord	NR	1×10^6 MSC cells	N/A	Cryoprotectant and PBS	Fresh-MSCs	*P*<0.05	*P*<0.05	↔	NS
Horiuchi et al., 2021	Osteoarthritis model in rats	Bioluminescence	9	Rat synovial MSCs	NR	1×10^6 MSC cells	N/A	PBS	Fresh-MSCs	NR	NR	↔	NS
		Tibia gross finding score	9	Rat synovial MSCs	NR	1×10^6 MSC cells	N/A	PBS	Fresh-MSCs	*P*<0.05	*P*<0.05	↔	NS
		Femur gross finding score	9	Rat synovial MSCs	NR	1×10^6 MSC cells	N/A	PBS	Fresh-MSCs	*P*<0.05	*P*<0.05	↔	NS
		Tibia OARSI score	6	Rat synovial MSCs	NR	1×10^6 MSC cells	N/A	PBS	Fresh-MSCs	*P*<0.05	*P*<0.05	↔	NS
		Femur OARSI score	6	Rat synovial MSCs	NR	1×10^6 MSC cells	N/A	PBS	Fresh-MSCs	NS	NS	↔	NS
Horie et al., 2021	Ventilator-Induced Lung Injury (VILI) model in rats	Arterial oxygenation	7	Human Umbilical Cord MSCs	NR	1 × 10^7 MSCs/kg	N/A	PBS	Fresh MSCs	*P*<0.001	*P*<0.001	↔	NS
		Static Lung Compliance	7	Human Umbilical Cord MSCs	NR	1 × 10^7 MSCs/kg	N/A	PBS	Fresh MSCs	*P*<0.01	*P*<0.01	↔	NS
		Wet:Dry Ratio	7	Human Umbilical Cord MSCs	NR	1 × 10^7 MSCs/kg	N/A	PBS	Fresh MSCs	*P*<0.05	*P*<0.05	↔	NS
		BAL Protein	7	Human Umbilical Cord MSCs	NR	1 × 10^7 MSCs/kg	N/A	PBS	Fresh MSCs	*P*<0.01	*P*<0.01	↔	NS
		BAL Cell count	7	Human Umbilical Cord MSCs	NR	1 × 10^7 MSCs/kg	N/A	PBS	Fresh MSCs	*P*<0.01	*P*<0.01	↔	NS
		BAL Neutrophil count	7	Human Umbilical Cord MSCs	NR	1 × 10^7 MSCs/kg	N/A	PBS	Fresh MSCs	*P*<0.05	*P*<0.05	↔	NS
		BAL IL-6 level	7	Human Umbilical Cord MSCs	NR	1 × 10^7 MSCs/kg	N/A	PBS	Fresh MSCs	NS	*P*<0.05	Frozen better	*P*<0.05
		BAL IL-1 level	7	Human Umbilical Cord MSCs	NR	1 × 10^7 MSCs/kg	N/A	PBS	Fresh MSCs	*P*<0.05	*P*<0.05	↔	NS
		% Airspace	4	Human Umbilical Cord MSCs	NR	1 × 10^7 MSCs/kg	N/A	PBS	Fresh MSCs	*P*<0.001	*P*<0.001	↔	NS

↔ indicates no statistically significant difference of Freshly-cultured and Cryopreserved MSCs.

NS indicates Not Significant- statistical analysis from individual studies did not yield significant difference between Freshly-cultured and Cryopreserved MSCs. NR = Not reported.

If direct comparison of Freshly-cultured vs. Cryopreserved MSC was not presented in the same graph by a study, the results and discussion sections of that study were used to judge efficacy of Freshly-cultured vs. Cryopreserved MSCs for the table above.

**Table 5.** Summary of similar in-vivo outcomes reported across studies.

Outcome Measure	Study	Unit of Measurement	Number of samples (n)	Fresh MSC Mean	Fresh MSC Std Dev	Frozen MSC Mean	Frozen MSC Std Dev
**Arterial Oxygenation0.128**	Curley et al., 2017	mmHg	8 to 10	217.77	77.93	242.75	84.14
	Devaney et al., 2015	mmHg	10	265.5	67.86	247.64	68.232
	Horie et al., 2020a	mmHg	8	73.084	11.526	69.148	9.222
	Horie et al., 2021	kPa	7	16.52	0.85	16.86	1.10
**Lung Compliance**	Curley et al., 2017	mL/mmHg	8 to 10	0.862	0.082	0.818	0.098
	Devaney et al., 2015	mL/mmHg	12	0.82264	0.132	0.765	0.128
	Horie et al., 2020a	mL/mmHg	8	0.55939	0.089	0.451	0.531
	Horie et al., 2021	mL/cmH2O	7	0.363	0.06	0.358	0.08
**Wet:Dry Lung Ratio**	Curley et al., 2017	Ratio	8 to 10	4.72779	0.188	4.77	0.157
	Horie et al., 2020a	Ratio	8	4.7643	0.074	4.94	0.294
	Horie et al., 2021	Ratio	7	5.21	0.36	5.32	0.42
**BAL IL-6 levels**	Devaney et al., 2015	pg/ml	12	348.93	207.5	363.22	142.5
	Horie et al., 2020a	pg/ml	8	224.67	119.86	181.51	126.72
	Horie et al., 2021	pg/ml	7	252.39	61.64	207.76	53.66
**% of Wound Contraction on D7**	Somal et al., 2017	Percentage	3	60.076	16.67	55.679	12.755
	Bharti et al., 2020	Percentage	5	16.104	1.062	14.521	2.123
	Rogulska et al., 2019	Percentage	14	51.402	5.741	52.069	4.94
**% of Wound Contraction on D14**	Somal et al., 2017	Percentage	3	96.374	0.85	89.937	5.103
	Bharti et al., 2020	Percentage	5	67.363	1.69	71.537	2.123
	Rogulska et al., 2019	Percentage	14	99.065	2.8	99.866	2.804
**% of Wound Contraction on D21**	Somal et al., 2017	Percentage	3	99.85	0.681	98.515	2.89
	Bharti et al., 2020	Percentage	5	84.141	1.93	89.457	1.769
**% of Wound Contraction on D28**	Somal et al., 2017	Percentage	3	100.433		100.288	0.681
	Bharti et al., 2020	Percentage	5	99.583	0.885	99.415	0.885

### Primary outcomes

#### In vivo pre-clinical efficacy outcomes

The 18 studies reported a total of 257 experiments and 101 distinct outcome measures related to our in vivo pre-clinical efficacy primary outcomes. Seventeen studies assessed composition of tissues (Cruz et al., 2015; Devaney et al., 2015; Gramlich et al., 2016; Salmenkari et al., 2019; Somal et al., 2017; Tan et al., 2019; Curley et al., 2017; Horiuchi et al., 2021; Horie et al., 2021; Yea et al., 2020; Bárcia et al., 2017; Bharti et al., 2020; Khan et al., 2019; Horie et al., 2020a; Lohan et al., 2018; Perlee et al., 2019; Rogulska et al., 2019), and 12 assessed organ dysfunction (Cruz et al., 2015; Devaney et al., 2015; Gramlich et al., 2016; Salmenkari et al., 2019; Curley et al., 2017; Horiuchi et al., 2021; Horie et al., 2021; Yea et al., 2020; Bárcia et al., 2017; Khan et al., 2019; Horie et al., 2020a; Horie et al., 2020b). Eleven of the 18 studies assessed protein expression and secretion (Cruz et al., 2015; Devaney et al., 2015; Salmenkari et al., 2019; Tan et al., 2019; Curley et al., 2017; Horiuchi et al., 2021; Khan et al., 2019; Horie et al., 2020a; Lohan et al., 2018; Perlee et al., 2019; Horie et al., 2020b) (Table 2)**.**

Of the 257 experiments, six outcomes were significantly different at the 0.05 level or less, with two that favoured freshly cultured and four that favoured cryopreserved MSCs (Table 4).

#### In vivo pre-clinical efficacy: function and composition of tissue

**Figure 2.**
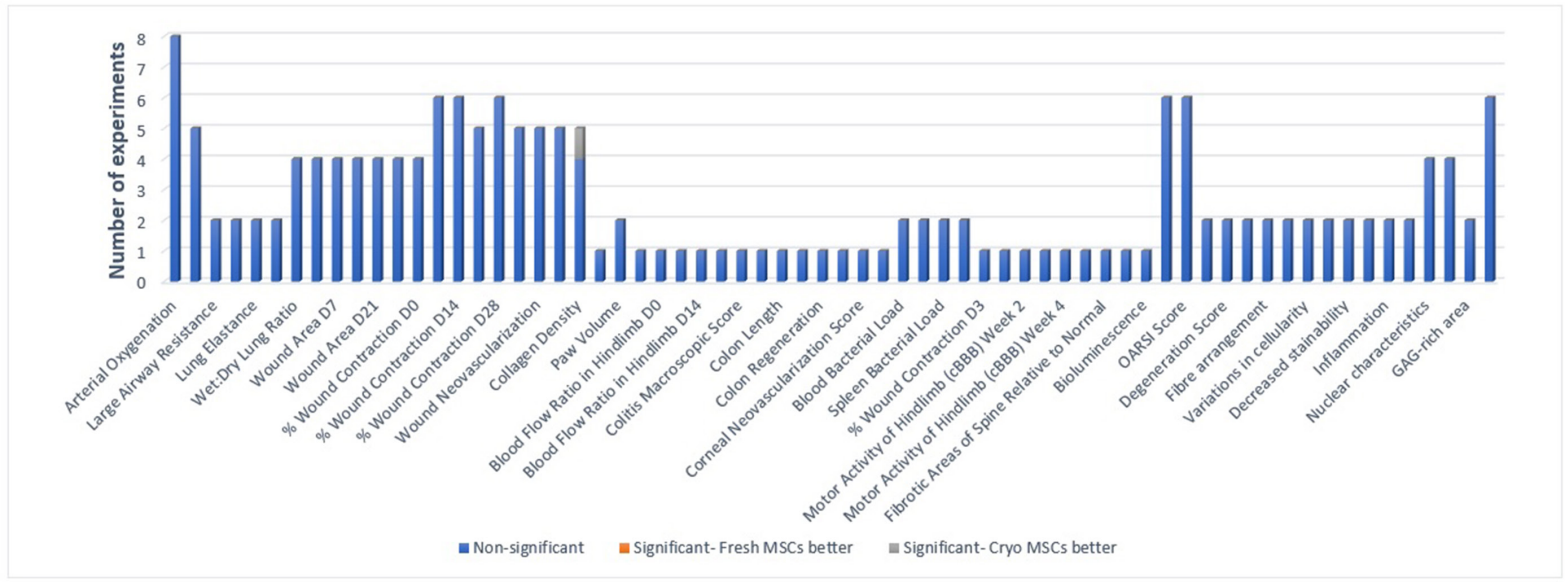
Primary in vivo outcomes. All the outcomes related to function and composition of tissues are presented below. Number of experiments represent the number of separate comparisons between freshly cultured and cryopreserved MSCs on surrogate measures of in vivo efficacy.

Seventeen studies reported organ dysfunction and/or composition of tissue outcomes and a total of 166 experiments were reported across the studies. Of the 116 experiments, only one reported a significant difference at the 0.05 level or less between the freshly cultured and cryopreserved MSC groups which favoured the cryopreserved group (Figure 2).

#### In vivo pre-clinical efficacy: protein (cytokine) expression and secretion

**Figure 3.**
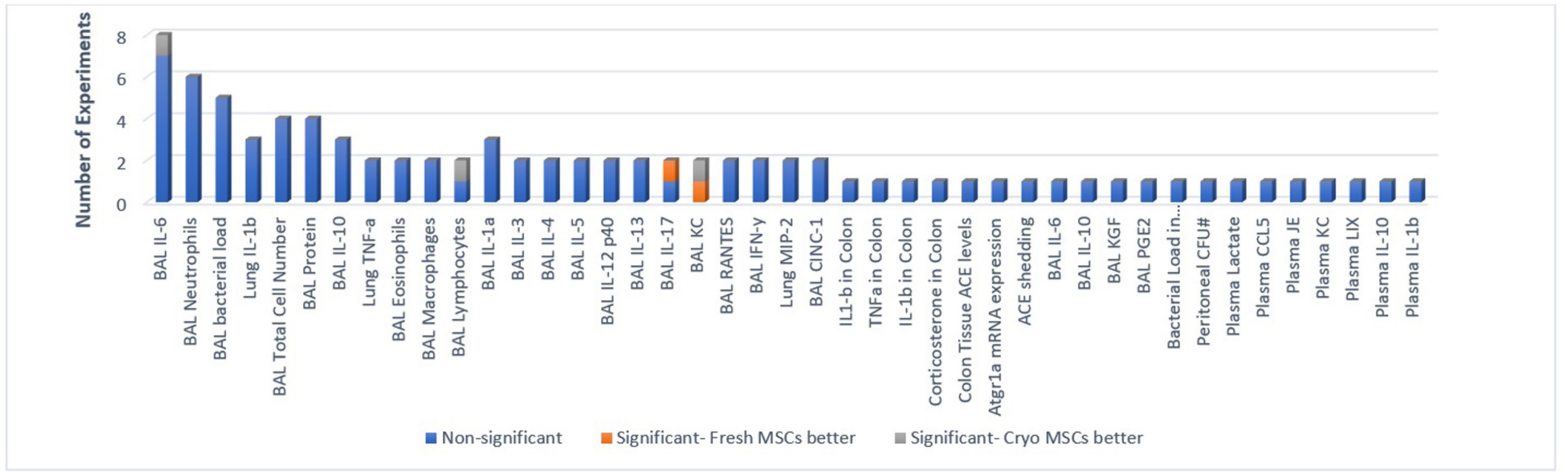
Primary in-vivo outcomes. All the outcomes related to protein (cytokine) expression and secretion are presented below. Number of experiments represent the number of separate comparisons between freshly cultured and cryopreserved MSCs on surrogate measures of in vivo efficacy.

Eleven studies reported protein expression and secretion outcomes, with total of 91 experiments reported across the studies. Five of the 91 experiments reported a statistically significant difference between freshly cultured and cryopreserved MSCs that were derived from one study (Cruz et al., 2015). Of the five experiments that demonstrated a significant difference at the 0.05 level or less, two favoured freshly cultured and three favoured cryopreserved MSCs (Figure 3).

### Secondary outcomes

#### In vitro potency outcomes

**Figure 4.**
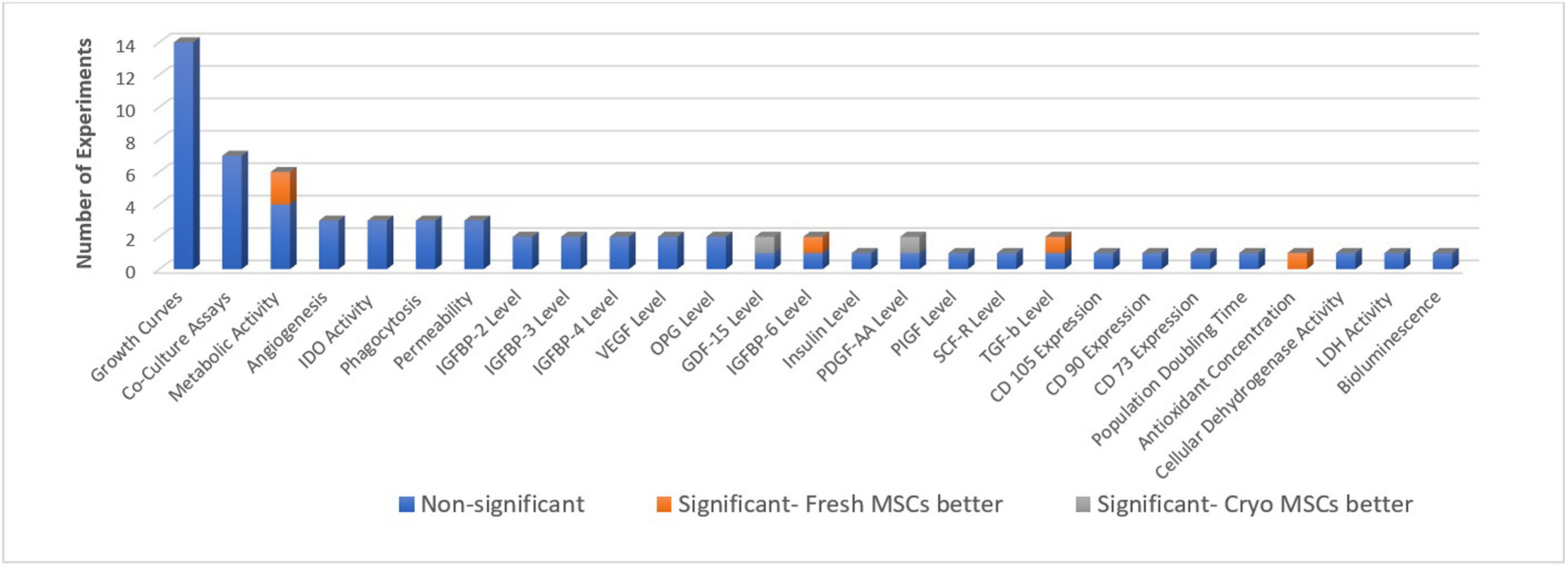
In-vitro potency outcomes. All the in-vitro reported outcomes are displayed below. Number of experiments represent the number of separate comparisons between freshly-cultured and cryopreserved MSCs on surrogate measures of in vivo efficacy.

Fifteen studies reported in vitro potency outcomes, including viability (Cruz et al., 2015; Devaney et al., 2015; Gramlich et al., 2016; Somal et al., 2017; Tan et al., 2019; Curley et al., 2017; Horiuchi et al., 2021; Bárcia et al., 2017; Bharti et al., 2020; Khan et al., 2019; Horie et al., 2020a; Lohan et al., 2018; Perlee et al., 2019; Rogulska et al., 2019; Horie et al., 2020b) with 68 experiments and 32 different outcome measures. All reported in vitro outcomes can be found in Table 6. Of the 68 experiments, 9 were significantly different at the 0.05 level or less, with 7 that favoured freshly cultured and 2 that favoured cryopreserved MSCs (Figure 4).

**Table 6.** In vitro outcomes where freshly cultured vs. cryopreserved MSCs were compared directly.

Study	Outcome	Assay Used	Number (n)	Type and Source of MSCs	Time of cell preparation without MSC (hr)	Time of outcome measurement from MSC intervention (hr)	Concentration of MSCs	Pre-Treatment of MSCs	Negative Control (NC)	Positive Control (PC)	p-value for Fresh MSCs vs. control	p-value for Frozen MSCs vs. control	Fresh or Frozen MSC more effective?	p-value for Fresh vs. Frozen comparison
Bárcia et al., 2017	Viability	Trypan Blue	Fresh/Cultured (12); cryo <1 yr(12); cryo >3 yrs (5)	Human Umbilical Cord MSCs	N/A	0	NR	Fresh/Cultured MSCs were cryopreserved and then cultured for up to 5 days	N/A	N/A	N/A	N/A	↔	NS
	Apoptosis	Annexin V (and flow cytometry)	N/A	Human Umbilical Cord MSCs	N/A	2	NR	Fresh/Cultured MSCs were cryopreserved and then cultured for up to 5 days	N/A	Cultured cells incubated with H_2_O_2_ (2 mmol/L) for 2 hr	NR	NR	↔	NS
	Angiogenesis: Number of master junctions (branching points)	Matrigel/Human umbilical vein endothelial cell (HUVEC) tube formation assay	2	Human Umbilical Cord MSCs	1	16	1 × 10^6^ cells	Fresh/Cultured MSCs were cryopreserved and then cultured for up to 5 days; fresh and cryo co-cultured in basal media	N/A	HUVEC in Basal Media and HUVECs in Basal media with VEGF (100 ng/mL)	NR	NR	↔	NS
	Angiogenesis: segment/tube length	Matrigel/Human umbilical vein endothelial cell (HUVEC) tube formation assay	2	Human Umbilical Cord MSCs	1	16	1 × 10^6^ cells	Fresh/Cultured MSCs were cryopreserved and then cultured for up to 5 days; fresh and cryo co-cultured in basal media	N/A	HUVEC in Basal Media and HUVECs in Basal media with VEGF (100 ng/mL)	NR	NR	↔	NS
	Angiogenesis:total mesh area	Matrigel/Human umbilical vein endothelial cell (HUVEC) tube formation assay	2	Human Umbilical Cord MSCs	1	16	1 × 10^6^ cells	Fresh/Cultured MSCs were cryopreserved and then cultured for up to 5 days; fresh and cryo co-cultured in basal media	N/A	HUVEC in Basal Media and HUVECs in Basal media with VEGF (100 ng/mL)	NR	NR	↔	NS
Gramlich et al., 2016	Viability	TUNEL staining via Apo-Direct Apoptosis Detection Kit	5	Human MSCs	N/A	24	30,000 MSCs	Both fresh and frozen cells were washed twice, resuspended in PBS and analyzed immediately or after 1 hr storage on wet ice	N/A	N/A	N/A	N/A	Fresh better	*P*<0.001
	Viability	TUNEL staining via Apo-Direct Apoptosis Detection Kit	5	Human MSCs	N/A	48	30,000 MSCs	Both fresh and frozen cells were washed twice, resuspended in PBS and analyzed immediately or after 1 hr storage on wet ice	N/A	N/A	N/A	N/A	Fresh better	*P*<0.001
	Viability	TUNEL staining via Apo-Direct Apoptosis Detection Kit	5	Human MSCs	N/A	72	30,000 MSCs	Both fresh and frozen cells were washed twice, resuspended in PBS and analyzed immediately or after 1 hr storage on wet ice	N/A	N/A	N/A	N/A	Fresh better	*P*=0.002
	Metabolic Activity (measured by XXT)	XTT Assay	6	Human MSCs	N/A	24	15,000 MSCs	N/A	N/A	N/A	N/A	N/A	↔	NS *P*=0.352
	Metabolic Activity (measured by XXT)	XTT Assay	6	Human MSCs	N/A	48	15,000 MSCs	N/A	N/A	N/A	N/A	N/A	↔	NS *P*=0.312
	Metabolic Activity (measured by XXT)	XTT Assay	6	Human MSCs	N/A	72	15,000MSCs	N/A	N/A	N/A	N/A	N/A	↔	NS *P*=0.971
	IDO activity: unstimulated MSC	Concentration of kynurenine in conditioned media	6	Human MSC	N/A	48	NR	N/A	N/A	N/A	N/A	N/A	↔	NS *P*=0.998
	IDO activity:MSC exposed to IFN-y	Concentration of kynurenine in conditioned media	6	Human MSC	N/A	48	NR	N/A	N/A	N/A	N/A	N/A	↔	NS *P*=0.099
	IDO activity: MSC exposed to IFN-y+TNF a	Concentration of kynurenine in conditioned media	6	Human MSC	N/A	48	NR	N/A	N/A	N/A	N/A	N/A	↔	NS *P*=0.951
	GDF-15: unstimulated	Human Growth Factor Array Q1	4	Human MSC	N/A	48	200,000 MSCs	N/A	N/A	Media Control	N/A	N/A	Frozen better	*P*=0.01
	GDF-15: stimulated with IFN-y/TNF-a	Human Growth Factor Array Q1	4	Human MSC	N/A	48	200,000 MSCs	N/A	N/A	Media Control	N/A	N/A	↔	NS *P*=0.99
	IGFBP-2: unstimulated	Human Growth Factor Array Q1	4	Human MSC	N/A	48	200,000 MSCs	N/A	N/A	Media Control	N/A	N/A	↔	NS *P*=0.32
	IGFBP-2: stimulated with IFN-y/TNF-a	Human Growth Factor Array Q1	4	Human MSC	N/A	48	200,000 MSCs	N/A	N/A	Media Control	N/A	N/A	↔	NS *P*=0.68
	IGFBP-3: unstimulated	Human Growth Factor Array Q1	4	Human MSC	N/A	48	200,000 MSCs	N/A	N/A	Media Control	N/A	N/A	↔	NS *P*=0.47
	IGFBP-3: stimulated with IFN-y/TNF-a	Human Growth Factor Array Q1	4	Human MSC	N/A	48	200,000 MSCs	N/A	N/A	Media Control	N/A	N/A	↔	NS *P*=0.75
	IGFBP-4: unstimulated	Human Growth Factor Array Q1	4	Human MSC	N/A	48	200,000 MSCs	N/A	N/A	Media Control	N/A	N/A	↔	NS *P*=0.39
	IGFBP-6: unstimulated	Human Growth Factor Array Q1	4	Human MSC	N/A	48	200,000 MSCs	N/A	N/A	Media Control	N/A	N/A	↔	NS *P*=0.69
	IGFBP-6: stimulated with IFN-y/TNF-a	Human Growth Factor Array Q1	4	Human MSC	N/A	48	200,000 MSCs	N/A	N/A	Media Control	N/A	N/A	Fresh better	*P*=0.03
	Insulin: stimulated with IFN-y/TNF-a	Human Growth Factor Array Q1	4	Human MSC	N/A	48	200,000 MSCs	N/A	N/A	Media Control	N/A	N/A	↔	NS *P*=0.71
	OPG: unstimulated	Human Growth Factor Array Q1	4	Human MSC	N/A	48	200,000 MSCs	N/A	N/A	Media Control	N/A	N/A	↔	NS *P*=0.39
	OPG: stimulated with IFN-y/TNF-a	Human Growth Factor Array Q1	4	Human MSC	N/A	48	200,000 MSCs	N/A	N/A	Media Control	N/A	N/A	↔	NS *P*=0.65
	PDGF-AA: unstimulated	Human Growth Factor Array Q1	4	Human MSC	N/A	48	200,000 MSCs	N/A	N/A	Media Control	N/A	N/A	↔	NS *P*=0.43
	PDGF-AA: stimulated with IFN-y/TNF-a	Human Growth Factor Array Q1	4	Human MSC	N/A	48	200,000 MSCs	N/A	N/A	Media Control	N/A	N/A	Frozen better	*P*=0.04
	PIGF: unstimulated	Human Growth Factor Array Q1	4	Human MSC	N/A	48	200,000 MSCs	N/A	N/A	Media Control	N/A	N/A	↔	NS *P*=0.83
	SCF R: stimulated with IFN-y/TNF-a	Human Growth Factor Array Q1	4	Human MSC	N/A	48	200,000 MSCs	N/A	N/A	Media Control	N/A	N/A	↔	NS *P*=0.06
	TGFb1: unstimulated	Human Growth Factor Array Q1	4	Human MSC	N/A	48	200,000 MSCs	N/A	N/A	Media Control	N/A	N/A	N/A	N/A
	TGFb1: stimulated with IFN-y/TNF-a	Human Growth Factor Array Q1	4	Human MSC	N/A	48	200,000 MSCs	N/A	N/A	Media Control	N/A	N/A	Fresh better	*P*=0.05
	VEGF: unstimulated	Human Growth Factor Array Q1	4	Human MSC	N/A	48	200,000 MSCs	N/A	N/A	Media Control	N/A	N/A	↔	NS *P*=0.30
	VEGF: stimulated with IFN-y/TNF-a	Human Growth Factor Array Q1	4	Human MSC	N/A	48	200,000 MSCs	N/A	N/A	Media Control	N/A	N/A	↔	NS *P*=0.96
Tan et al., 2019	Viability	Trypan Blue	NR	Human BM	N/A	0	NR	N/A	N/A	N/A	N/A	N/A	↔	NS
	Viability	Trypan Blue	NR	Human BM	N/A	2	NR	N/A	N/A	N/A	N/A	N/A	Fresh better	*P*<0.05
	Viability	Trypan Blue	NR	Human BM	N/A	4	NR	N/A	N/A	N/A	N/A	N/A	↔	NS
	Viability	Trypan Blue	NR	Human BM	N/A	6	NR	N/A	N/A	N/A	N/A	N/A	↔	NS
	Viability (Viable Cells)	Annexin V+Propidium iodide (AV/PI)	NR	Human BM	N/A	0	NR	N/A	N/A	N/A	N/A	N/A	↔	NS
	Viability (Viable Cells)	Annexin V+Propidium iodide (AV/PI)	NR	Human BM	N/A	2	NR	N/A	N/A	N/A	N/A	N/A	↔	NS
	Viability (Viable Cells)	Annexin V+Propidium iodide (AV/PI)	NR	Human BM	N/A	4	NR	N/A	N/A	N/A	N/A	N/A	↔	NS
	Viability (Viable Cells)	Annexin V+Propidium iodide (AV/PI)	NR	Human BM	N/A	6	NR	N/A	N/A	N/A	N/A	N/A	Fresh better	*P*<0.05
	Viability(Early apoptotic cells)	Annexin V+Propidium iodide (AV/PI)	NR	Human BM	N/A	0	NR	N/A	N/A	N/A	N/A	N/A	↔	NS
	Viability(Early apoptotic cells)	Annexin V+Propidium iodide (AV/PI)	NR	Human BM	N/A	2	NR	N/A	N/A	N/A	N/A	N/A	↔	NS
	Viability(Early apoptotic cells)	Annexin V+Propidium iodide (AV/PI)	NR	Human BM	N/A	4	NR	N/A	N/A	N/A	N/A	N/A	↔	NS
	Viability(Early apoptotic cells)	Annexin V+Propidium iodide (AV/PI)	NR	Human BM	N/A	6	NR	N/A	N/A	N/A	N/A	N/A	Fresh better	*P*<0.05
	Viability (Late apoptotic cells)	Annexin V+Propidium iodide (AV/PI)	NR	Human BM	N/A	0	NR	N/A	N/A	N/A	N/A	N/A	↔	NS
	Viability (Late apoptotic cells)	Annexin V+Propidium iodide (AV/PI)	NR	Human BM	N/A	2	NR	N/A	N/A	N/A	N/A	N/A	↔	NS
	Viability (Late apoptotic cells)	Annexin V+Propidium iodide (AV/PI)	NR	Human BM	N/A	4	NR	N/A	N/A	N/A	N/A	N/A	Fresh better	*P*<0.05
	Viability (Late apoptotic cells)	Annexin V+Propidium iodide (AV/PI)	NR	Human BM	N/A	6	NR	N/A	N/A	N/A	N/A	N/A	Fresh better	*P*<0.05
	Phagocytosis	PBMCs pre-treated with LPS the co-culture with MSC at ratio of 1:5 for 24 hr	3–6	Human BM MSC: Donor 1	N/A	24	NR	N/A	Naive PBMC	LPS treated PBMC	PC: *P*<0.0001	PC: *P*<0.0001	↔	NS
	Phagocytosis	PBMCs pre-treated with LPS the co-culture with MSC at ratio of 1:5 for 24 hr	3–6	Human BM MSC: Donor 2	N/A	24	NR	N/A	Naive PBMC	LPS treated PBMC	NS	NS	↔	NS
	Phagocytosis	PBMCs pre-treated with LPS the co-culture with MSC at ratio of 1:5 for 24 hr	3–6	Human BM MSC: Donor 3	N/A	24	NR	N/A	Naive PBMC	LPS treated PBMC	PC: *P*<0.001	PC: *P*<0.001	↔	NS
	Permeability	Endothelial cell (EC) treated with LPS for 6 hr then co-culture with MSC for 24 hr at ratio of 1:2 followed by adding FITC-dextran to the transwell insert	NR	Human BM MSC: Donor 1	N/A	24	NR	N/A	Non-treated EC	LPS treated EC	PC: *P*<0.01	PC: *P*<0.01	↔	NS
	Permeability	Endothelial cell (EC) treated with LPS for 6 hr then co-culture with MSC for 24 hr at ratio of 1:2 followed by adding FITC-dextran to the transwell insert	NR	Human BM MSC: Donor 2	N/A	24	NR	N/A	Non-treated EC	LPS treated EC	PC: *P*<0.01	PC: *P*<0.01	↔	NS
	Permeability	Endothelial cell (EC) treated with LPS for 6 hr then co-culture with MSC for 24 hr at ratio of 1:2 followed by adding FITC-dextran to the transwell insert	NR	Human BM MSC: Donor 3	N/A	24	NR	N/A	Non-treated EC	LPS treated EC	PC: *P*<0.001	PC: *P*<0.001	↔	NS
Bharti et al., 2020	Growth Curve	Countess automated cell counter	NR	Canine BM	N/A	24	1 × 10^4^ cells/ml	Frozen cells were thawed in distilled water at 36 °C for 45–60 s then enzymatically detached from mesh and added in re-warmed media with 15% FBS and washed twice at 1200 rpm for 5 min	N/A	N/A	N/A	N/A	↔	NS
	Growth Curve	Countess automated cell counter	NR	Canine BM	N/A	48	1 × 10^4^ cells/ml	Frozen cells were thawed in distilled water at 36 °C for 45–60 s then enzymatically detached from mesh and added in re-warmed media with 15% FBS and washed twice at 1200 rpm for 5 min	N/A	N/A	N/A	N/A	↔	NS
	Growth Curve	Countess automated cell counter	NR	Canine BM	N/A	72	1 × 10^4^ cells/ml	Frozen cells were thawed in distilled water at 36 °C for 45–60 s then enzymatically detached from mesh and added in re-warmed media with 15% FBS and washed twice at 1200 rpm for 5 min	N/A	N/A	N/A	N/A	↔	NS
	Growth Curve	Countess automated cell counter	NR	Canine BM	N/A	96	1 × 10^4^ cells/ml	Frozen cells were thawed in distilled water at 36 °C for 45–60 s then enzymatically detached from mesh and added in re-warmed media with 15% FBS and washed twice at 1200 rpm for 5 min	N/A	N/A	N/A	N/A	↔	NS
	Growth Curve	Countess automated cell counter	NR	Canine BM	N/A	120	1 × 10^4^ cells/ml	Frozen cells were thawed in distilled water at 36 °C for 45–60 s then enzymatically detached from mesh and added in re-warmed media with 15% FBS and washed twice at 1200 rpm for 5 min	N/A	N/A	N/A	N/A	↔	NS
	Growth Curve	Countess automated cell counter	NR	Canine BM	N/A	144	1 × 10^4^ cells/ml	Frozen cells were thawed in distilled water at 36 °C for 45–60 s then enzymatically detached from mesh and added in re-warmed media with 15% FBS and washed twice at 1200 rpm for 5 min	N/A	N/A	N/A	N/A	↔	NS
	Growth Curve	Countess automated cell counter	NR	Canine BM	N/A	168	1 × 10^4^ cells/ml	Frozen cells were thawed in distilled water at 36 °C for 45–60 s then enzymatically detached from mesh and added in re-warmed media with 15% FBS and washed twice at 1200 rpm for 5 min	N/A	N/A	N/A	N/A	↔	NS
	Growth Curve	Countess automated cell counter	NR	Canine BM	N/A	192	1 × 10^4^ cells/ml	Frozen cells were thawed in distilled water at 36 °C for 45–60 s then enzymatically detached from mesh and added in re-warmed media with 15% FBS and washed twice at 1200 rpm for 5 min	N/A	N/A	N/A	N/A	↔	NS
	Growth Curve	Countess automated cell counter	NR	Canine BM	N/A	216	1 × 10^4^ cells/ml	Frozen cells were thawed in distilled water at 36 °C for 45–60 s then enzymatically detached from mesh and added in re-warmed media with 15% FBS and washed twice at 1200 rpm for 5 min	N/A	N/A	N/A	N/A	↔	NS
	Growth Curve	Countess automated cell counter	NR	Canine BM	N/A	240	1 × 10^4^ cells/ml	Frozen cells were thawed in distilled water at 36 °C for 45–60 s then enzymatically detached from mesh and added in re-warmed media with 15% FBS and washed twice at 1200 rpm for 5 min	N/A	N/A	N/A	N/A	↔	NS
	Growth Curve	Countess automated cell counter	NR	Canine BM	N/A	264	1 × 10^4^ cells/ml	Frozen cells were thawed in distilled water at 36 °C for 45–60 s then enzymatically detached from mesh and added in re-warmed media with 15% FBS and washed twice at 1200 rpm for 5 min	N/A	N/A	N/A	N/A	↔	NS
	Growth Curve	Countess automated cell counter	NR	Canine BM	N/A	288	1 × 10^4^ cells/ml	Frozen cells were thawed in distilled water at 36 °C for 45–60 s then enzymatically detached from mesh and added in re-warmed media with 15% FBS and washed twice at 1200 rpm for 5 min	N/A	N/A	N/A	N/A	↔	NS
	Growth Curve	Countess automated cell counter	NR	Canine BM	N/A	312	1 × 10^4^ cells/ml	Frozen cells were thawed in distilled water at 36 °C for 45–60 s then enzymatically detached from mesh and added in re-warmed media with 15% FBS and washed twice at 1200 rpm for 5 min	N/A	N/A	N/A	N/A	↔	NS
	CD 105 expression	Antibody assay	NR	Canine BM	N/A	Overnight	NR	Primary antibodies (1:100 dilutions) were used for localizing different markers (CD73, CD90, CD105, CD34) with an overnight incubation period at 4 °C.	N/A	N/A	N/A	N/A	↔	NS
	CD 90 expression	Antibody assay	NR	Canine BM	N/A	Overnight	NR	Primary antibodies (1:100 dilutions) were used for localizing different markers (CD73, CD90, CD105, CD34) with an overnight incubation period at 4 °C.	N/A	N/A	N/A	N/A	↔	NS
	CD 73 expression	Antibody assay	NR	Canine BM	N/A	Overnight	NR	Primary antibodies (1:100 dilutions) were used for localizing different markers (CD73, CD90, CD105, CD34) with an overnight incubation period at 4 °C.	N/A	N/A	N/A	N/A	↔	NS
	Population Doubling Time	N/A	NR	Canine BM	N/A	N/A	1 × 10^4^ cells/ml	N/A	N/A	N/A	N/A	N/A	↔	NS
Rogulska et al., 2019	Metabolic Activity/Proliferation rate	Alamar Blue	3	Human Adipose	N/A	48	NR	MSCs culture in PS1D-based gel	N/A	N/A	N/A	N/A	Fresh better	*P*<0.05
	Metabolic Activity/Proliferation rate	Alamar Blue	3	Human Adipose	N/A	96	NR	MSCs culture in PS1D-based gel	N/A	N/A	N/A	N/A	Fresh better	*P*<0.05
	Metabolic Activity/Proliferation rate	Alamar Blue	3	Human Adipose	N/A	144	NR	MSCs culture in PS1D-based gel	N/A	N/A	N/A	N/A	↔	NS
	Viability	Alamar Blue	3	Human Adipose	N/A	24	NR	N/A	N/A	N/A	N/A	N/A	Fresh better	*P*<0.05
Khan et al., 2019	Antioxidant Concentration (2 fresh groups:GFP-MSC and HO-1 MSC)	Antioxidant Assay	6	Canine adipose	NR	NR	NR	Lentivirus-mediated GFP and HO-1 gene insertion into Ad-MSCs	N/A	N/A	N/A	N/A	Fresh better	*P*<0.05
Yea et al., 2020	Viability	Trypan Blue	6	Human Umbilical Cord	0	0, 2, 4, 24, 48 hr	1×10^4^ cells/well	None	N/A	N/A	N/A	N/A	↔	NS
	Viability	Water-soluble tetrazolium salt (WST) assay	6	Human Umbilical Cord	0	0, 2, 4, 24, 48 hr	1×10^4 cells/well	None	N/A	N/A	N/A	N/A	↔	NS
	Population Doubling Time	Cell counting	6	Human Umbilical Cord	0	4, 8, 12, 16, 20 days	3×10^3 cells/cm^2	None	N/A	N/A	N/A	N/A	↔	NS
Horiuchi et al., 2021	Biolumnescence	IVIS Lumina XRMS series III instrument (SPI, Tokyo, Japan)	4	Rat Synovial MSCs	0	Same day	Varying concentrations	None	N/A	N/A	N/A	N/A	↔	NS

N/A = Not applicable (e.g. if the experiment set up did not include a particular variable). NR = Not reported (e.g. if a particular variable was part of the experiment set up but not explicitly reported on in results section or graph).

#### In vitro potency: protein (cytokine) expression and secretion

A total of four studies (Gramlich et al., 2016; Horiuchi et al., 2021; Bharti et al., 2020; Khan et al., 2019) reported in vitro protein (cytokine) expression and secretion outcomes. Of the 33 experiments, five demonstrated a significant difference at the 0.05 level or less, with two favouring cryopreserved and three favouring freshly cultured MSCs (Table 5).

#### In vitro potency: co-culture assays

Three studies reported in vitro co-culture assay outcomes (7 separate experiments) to assess the impact of MSCs on responder cell proliferation (Gramlich et al., 2016; Tan et al., 2019; Bárcia et al., 2017). All three studies used PBMCs (peripheral blood mononuclear cell) activated with CD3 and CD28 as the responder cells. The studies employed variable MSC:Responder cell ratios and duration of culture. All three studies found no significant difference in potency for cryopreserved as compared to freshly-cultured MSCs at varying concentrations of MSCs to responder cells (Table 7).

**Table 7.** Summary of all in vitro PBMC Proliferation assays from included studies.

Study	MSCs Used	Solution	Addition to solution	Responder Cells	Fresh vs. Frozen Comparison	Duration of Culture	Proliferation Measurement	Ratio (MSC:Responder Cells)					
								1:1	1:3	1:6	1:10	1:12	1:50
Bárcia et al., 2017	Cultured and Freshly-thawed MSCs were irradiated with 50 Gy prior to use	RPMI	5% HEPES, 5% Pen-Strep, 5% NaPyr and 5% human serum	PBMC stimulated with anti-CD3, anti-CD28, and IL-2.	Yes	16 hr	Percentage of T cells proliferation/ suppression	Yes			Yes		Yes
Gramlich et al., 2016	Cultured and Freshly-thawed MSCs	RPMI	10% (v/v) FBS, 1% (v/v) Penicillin/Streptomycin, and 1% (v/v) L-glutamine	PBMC stimulated with 250,000 Human T-activator CD3+/D28+Dynabeads	Yes	144 hr	CFSE Cell Proliferation Kit		Yes	Yes		Yes	
Tan et al., 2019	Cultured and Freshly-thawed MSCs	NR	NR	PBMC stimulated with Dynabeads Human T-Activator CD3/CD28	Yes	120 hr			Yes				

### Viability

**Figure 5.**
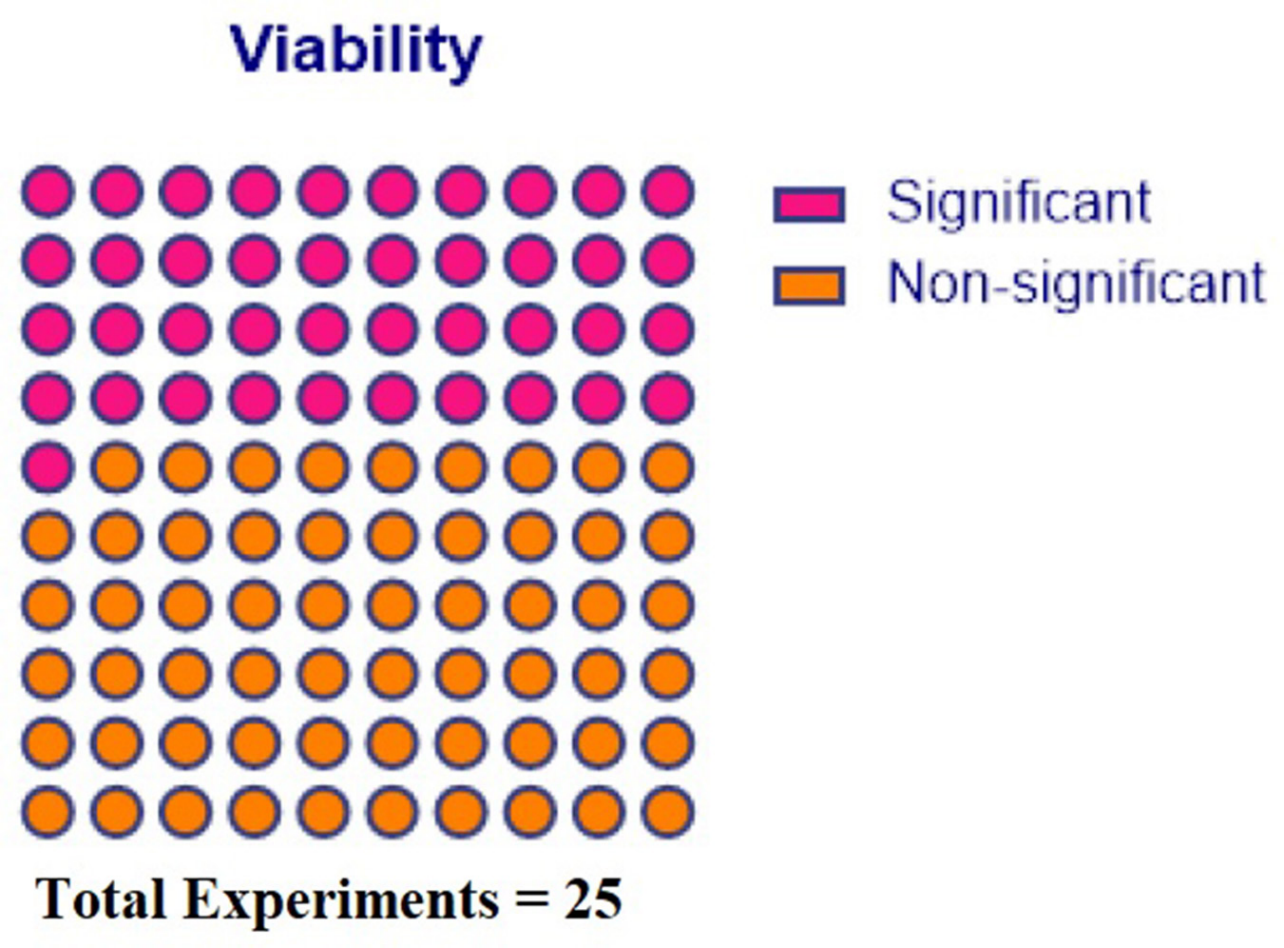
Comparison of viability. Experiments where viability at varying time points of freshly-cultured and cryopreserved MSCs were compared directly are presented below.

Seventeen studies (Cruz et al., 2015; Devaney et al., 2015; Gramlich et al., 2016; Somal et al., 2017; Tan et al., 2019; Curley et al., 2017; Horiuchi et al., 2021; Horie et al., 2021; Yea et al., 2020; Bárcia et al., 2017; Bharti et al., 2020; Khan et al., 2019; Horie et al., 2020a; Lohan et al., 2018; Perlee et al., 2019; Rogulska et al., 2019; Horie et al., 2020b) reported post-thaw viability of cryopreserved MSCs, the range was from 60% to 98% across various time points since thawing. The viability of freshly cultured MSCs ranged from 91% to 99%, also assessed at various time points. Only seven studies reported on 25 viability experiments which compared viability directly between freshly cultured and cryopreserved MSCs (Gramlich et al., 2016; Somal et al., 2017; Tan et al., 2019; Horiuchi et al., 2021; Horie et al., 2021; Yea et al., 2020; Bárcia et al., 2017) Of the 25 experiments, 9 (36%) favoured freshly cultured MSCs (Figure 5).

## Discussion

Our study is the first comprehensive pre-clinical systematic review to examine the effect of cryopreservation on the in vivo efficacy and in vitro potency of MSCs in animal models of inflammation. Across the 18 included studies, our review found that 251 out of 257 (97.6%) of the in vivo pre-clinical efficacy outcomes demonstrated no statistically significant differences between cryopreserved and freshly cultured MSCs at a p value of<0.05*.* When evaluating the results of a large, heterogeneous group of studies with different outcome measures comparing freshly cultured versus cryopreserved MSCs for efficacy and potency, it is useful to compare the results to what one would expect to see if (a) there were truly no difference or if (b) there truly were a difference. In the former case, where all differences would be due exclusively to Type I error, we would expect to see roughly 5% of the p-values as statistically significant. Furthermore, when a difference was statistically significant, we would expect it to be equally likely to favor freshly cultured versus cryopreserved or vice versa. In the latter case, where there truly is a difference, we would expect to see more than 5% of the p-values of all experiments as statistically significant and a strong concordance in the sense that most would favor the same group. We argue that our results for in vivo preclinical efficacy are consistent with pure Type 1 error (2.6% were statistically significant with roughly half favoring freshly cultured and half favoring cryopreserved MSCs). For in vitro potency, the results are somewhat less clear cut. We found 13% (95% Confidence Interval: 5–21%) were significantly different; 7 favored freshly cultured and 2 favored cryopreserved MSCs. Given that the confidence interval for the rate of statistical significance does not exclude 5% and that 2 of the 9 significant results favored cryopreserved MSCs, it does not represent strong evidence of a significant difference in in vitro potency. In terms of viability, the evidence supports reduced viability in cryopreserved versus freshly cultured MSCs, which is in keeping with previously published studies (Eaker et al., 2013; Robb et al., 2019).

Cryopreservation under safe and quality-controlled conditions remains critical for future real-world applications of MSC therapies (Abazari et al., 2017) by easing the logistical burden of supplying freshly cultured MSCs, enabling quality control and standardization of the cell preparation, and to facilitate the logistical transport of cellular products to hospitals. Some studies have shown that cryopreservation does not negatively impact MSCs; even if stored in cryopreservation for up to 23–24 years (Shen et al., 2012; Badowski et al., 2014; Marquez-Curtis et al., 2015). However, other studies have demonstrated mixed effects with both short-term and long-term cryopreservation (Dariolli et al., 2013; Kotobuki et al., 2005). Notably, most of these studies lack a clear assessment of MSC in vivo function. A recent systematic review of 41 in vitro studies that examined bone-marrow-derived MSCs (BM-MSCs) demonstrated that MSC cell morphology, marker expression, proliferation potential and tri-lineage differentiation capability were unaffected by stresses imposed by freezing and thawing, whereas viability, attachment to plasticware and migration, genomic stability and paracrine function of MSCs demonstrated conflicting results (Bahsoun et al., 2019). Out of their included 41 studies, only eight studied MSCs’ immune function (88% conducted co-culture assays) post-thaw with four studies concluding a negative effect and four concluding no effect of cryopreservation on MSC in vitro immune function. Interestingly, this review found that the immediate post-thaw viability varied from about 50% to 100% among the included studies; 16 studies reported no change in viability immediately after thawing and 10 studies reported significantly lower viability (Bahsoun et al., 2019).

Cryopreserved MSCs have a higher percentage of apoptotic cells than MSCs from fresh cultures (Haack-Sørensen and Kastrup, 2011). Many factors could contribute to the diminished viability and functionality of cryopreserved MSCs, including the source of MSCs, rate of cooling, storage temperature and period, method of recovery from cryopreservation, and the cryoprotectants used (Marquez-Curtis et al., 2015). Cryopreserved MSCs are commonly frozen in 5–10% DMSO and or fetal bovine serum (FBS) (Liu et al., 2010; Rowley et al., 1999), but there are disadvantages of using these agents. DMSO is used extensively as a cryopreservation agent in the autologous hematopoietic stem cell transplant population and may be toxic at higher concentrations (Alessandrino et al., 1999). Adverse events have been associated with DMSO (most common are nausea, vomiting, weakness) (Mitrus et al., 2018) but a recent systematic review that examined safety of MSCs in randomized controlled trials (RCTs) found no serious adverse event safety signals for freshly cultured versus cryopreserved MSCs (Thompson et al., 2020). Furthermore, the use of animal proteins from FBS may theoretically increase the risk of transferring infectious agents or stimulating unwanted immunological responses. Despite the continued search for the most optimal cryoprotectant, no consensus has been developed on the safest type and concentration of cryoprotectant to use (Galipeau and Sensébé, 2018). Optimizing the rate of cooling is as important as the thawing process, both of which can further contribute to cell injury. Apoptotic and necrotic pathways are activated in these cells 6–48 h post-thaw in response to low temperature exposure (Chinnadurai et al., 2016; Baust et al., 2009). Remarkably, many studies demonstrate that MSCs, isolated from diverse sources, cryopreserved using various cooling rates, in the presence of different cryoprotectants, stored for various lengths of time, and at various sub-zero temperatures still retain their biological properties post-thaw except for viability (Marquez-Curtis et al., 2015). Viability of MSCs is considered an important indicator of cryopreservation success where at least 90% viability for fresh MSC product and 70% viability for cryopreserved MSC product are considered the benchmark for pre-clinical application (Robb et al., 2019). One provocative study found that recipient cytotoxic cell activity causing apoptosis of infused MSCs or infusion of ex-vivo apoptotic MSCs and suggested it is one of the proposed mechanisms of immunomodulation for MSCs and the lower viability (or increased number of apoptotic cells) may in fact play a positive role in reducing the host inflammatory state (Galleu et al., 2017). In a safety systematic review of MSC randomized trials, only 52% and 14.5% reported on viability and potency respectively (Thompson et al., 2020). Our systematic review also found that 13 of 18 included studies received an “unclear” risk of bias in 5 out of 10 domains of the SYRCLE risk of bias tool due to insufficient and unclear reporting of important variables (eg. cryopreservation process, storage conditions, blinding, etc.). Due to the importance of reporting risk of bias elements as well as the cryopreservation and thaw process that could impact MSC quantity, quality, and efficacy, interpretation of MSC research studies remains limited. We strongly encourage the standardized reporting of these parameters by authors, reviewers, and journal editors as markers of reporting quality and to enhance transparency, reproducibility, and interpretation of MSC research studies.

From the perspective of clinical research and potential efficacy of cryopreserved MSCs, a phase III randomized clinical trial that examined whether a cryopreserved MSC product, PROCHYMAL (Remestemcel-L), or placebo compared to standard second line therapies alone in children with acute graft-versus-host disease (aGVHD) showed that high risk patients were more likely to have a partial response at 28 days with Remestemcel. Furthermore, a recently published systematic review that examined 55 randomized trials which used a MSC product versus control/usual care not only suggested evidence for safety of cryopreserved MSCs but also potential efficacy. Of the 15 trials that studied a cryopreserved product, 5 of them (33%) found significant differences favoring cryopreserved MSCs in either the primary or secondary endpoints (Kebriaei et al., 2020).

There are several strengths in this current systematic review. First, we have published our protocol which includes a transparent search strategy, pre-defined classifications for cryopreserved and freshly cultured MSCs and outcome measures, and minimal exclusion criteria. Ours is the first comprehensive systematic review assessing the in vivo efficacy of cryopreserved MSCs when directly compared to freshly cultured MSCs in animal models of inflammation. All variables and experimental details were collected and summarized systematically. Given the breadth and variety of in vivo and in vitro outcome measures, we report our data by considering each experiment where cryopreserved and freshly cultured MSCs are compared as an individual hypothesis test. Our review provides the totality of the existing pre-clinical evidence base, and we hope it will provide additional rationale for considering a cryopreserved MSC product for use in pre-clinical studies and clinical trials, and help identify research gaps for future related research (Galipeau and Sensébé, 2018).

Our study did have some limitations. Given our emphasis on including studies that examined MSC in vivo efficacy, we excluded all studies that only conducted in vitro studies. This led to a significant number of cryopreserved MSC studies being excluded and hence, our in vitro outcome reporting may be incomplete. However, when considering whether cryopreserved MSCs may be efficacious in clinical settings, pre-clinical in vivo efficacy outcomes might be more convincing than in vitro studies alone. Most of the preclinical studies did not provide sufficient information to adequately perform the SYRCLE risk of bias assessment, resulting in unclear reporting in at least three bias domains or more in all but one study, despite our attempts to contact authors to obtain further study details. Our ability to conduct meta-analyses on our primary outcome measures and according to subgroups was significantly limited by the heterogeneity of animal models included and breadth of outcomes measured. Finally, it is possible that other important in vivo pre-clinical efficacy or in vitro potency outcomes were not reported in our review. However, we designed and then conducted a systematic and transparent search using a pre-published protocol to enhance transparency and reproducibility, and to ensure we captured the totality of the evidence according to our study question. Questions remain related to MSC mechanisms of action in response to different immune stimuli, such as the effect of xenotransplanation. Further research to understand where there may be differences in effects of syngeneic MSCs as compared to xenogenic MSCs in models of inflammatory diseases related to HLA stimulation/expression, co-stimulatory molecules, paracrine factors, and species-specific cytokines and receptors may assist successful translation in human clinical trials (Prockop and Lee, 2017). Our review reported pre-dominantly on different biological outcome measures which does not provide a measure of overall animal health in a given inflammatory animal model. However, certain biological outcomes may be part of the mechanistic/causal pathway related to the disease (in the animal and humans) and may be considered as important surrogates for overall health. These biological outcomes in pre-clinical studies may also help to inform the exploration of them as predictive or prognostic variables in human clinical trials.

### Conclusions

Our study provides a comprehensive systematic review of pre-clinical studies comparing cryopreserved versus freshly cultured MSCs in animal models of inflammation. Our findings suggest that for the majority of outcomes measured in this review, cryopreservation does not negatively impact in vivo efficacy or in vitro potency of MSCs. With our systematic summary of the current evidence base, we hope it may provide MSC basic and research scientists additional rationale for considering a cryopreserved MSC product for use in pre-clinical studies and clinical trials, and help identify research gaps for future MSC-related research. We also strongly encourage the standardized reporting of important parameters related to risk of bias, MSC processing characteristics (e.g. cryopreservation and thawing protocols), storage conditions, viability, and potency as markers of study quality and to enhance transparency, reproducibility, and interpretation of MSC research studies.

## Data Availability

All data generated or analyzed in our review are provided in the attached tables and figures.

## Additional files

Supplementary file 1. Search strategy.

Supplementary file 2. PRISMA checklist.

Supplementary file 3. AGREE-II tool prompting questions.

Supplementary file 4. Data collection items.

MDAR checklist
