# Supplementary material for: Comparison of freshly cultured versus cryopreserved mesenchymal stem cells in animal models of inflammation: A pre-clinical systematic review
Source: eLife. 2022 Jul 15;11:e75053. doi: 10.7554/eLife.75053 (PMC9286731; doi:10.7554/eLife.75053)
Supplement: Supplementary file 1. [file elife-75053-supp1.docx]

Database: Ovid MEDLINE(R) ALL <1946 to June 05, 2020>

Search Strategy:

--------------------------------------------------------------------------------

1 Mesenchymal Stromal Cells/ (36546)

2 Mesenchymal Stem Cell Transplantation/ (11510)

3 Multipotent Stem Cells/ (3055)

4 (mesenchymal adj3 (cell* or stem or stromal or progenitor or multipotent or bone marrow or adipose or placenta*)).tw. (68307)

5 ((multipotent or multi-potent) adj (stroma* cell* or stem cell* or progenitor cell*)).tw,kw. (2504)

6 (marrow stroma* adj2 cell*).tw,kw. (7526)

7 (mesenchymal or marrow strom* or strom* cell*).kw,kw. (1066)

8 or/1-7 (83663)

9 exp Cryopreservation/ (36899)

10 cryoconserv*.tw,kw. (215)

11 cryopreserv*.tw,kw. (23932)

12 (fresh* adj5 thaw*).tw. (1731)

13 (freez* adj3 thaw*).tw. (12037)

14 (fresh cell* or continuously cultur*).tw,kw. (1238)

15 (thaw* or freez*).kw. (3536)

16 (fresh* adj3 cultur*).tw. (3662)

17 or/9-16 (62921)

18 8 and 17 (1027)

19 (2019062* or 2019063* or 201907* or 201908* or 201909* or 20191* or 2020*).dt. (1292861)

20 18 and 19 (81)

Database: Embase Classic+Embase <1947 to 2020 June 05>

Search Strategy:

--------------------------------------------------------------------------------

1 exp mesenchymal stem cell/ (59136)

2 mesenchymal stem cell transplantation/ (11420)

3 mesenchymal stroma cell/ (13029)

4 (mesenchymal adj3 (cell* or stem or stromal or progenitor or multipotent or bone marrow or adipose or placenta*)).tw. (97774)

5 ((multipotent or multi-potent) adj (stroma* cell* or stem cell* or progenitor cell*)).tw. (3354)

6 (marrow stroma* adj2 cell*).tw. (10352)

7 or/1-6 (120050)

8 cryopreservation/ (41644)

9 cryopreserv*.tw. (34469)

10 (freez* adj3 thaw*).tw. (15609)

11 cryoconserv*.tw. (327)

12 (fresh* adj5 thaw*).tw. (2481)

13 (fresh* adj3 cultur*).tw. (4745)

14 fresh cell*.tw. (1255)

15 continuously cultured.tw. (375)

16 or/8-15 (70752)

17 7 and 16 (1742)

18 conference abstract.pt. (3810714)

19 17 not 18 (1210)

20 (2019062* or 2019063* or 201907* or 201908* or 201909* or 20191* or 2020*).dc. (2046349)

21 19 and 20 (126)

**Web of Science – June 8, 2020**

# 9 **227**

#6 AND #3

Refined by: [excluding] DOCUMENT TYPES: ( MEETING ABSTRACT ) AND PUBLICATION YEARS: ( 2020 OR 2019 )

Indexes=SCI-EXPANDED, SSCI, A&HCI, CPCI-S, CPCI-SSH, ESCI Timespan=All years

# 8 1,600

#6 AND #3

Refined by: [excluding] DOCUMENT TYPES: ( MEETING ABSTRACT )

Indexes=SCI-EXPANDED, SSCI, A&HCI, CPCI-S, CPCI-SSH, ESCI Timespan=All years

#7 1,669

#6 AND #3

Indexes=SCI-EXPANDED, SSCI, A&HCI, CPCI-S, CPCI-SSH, ESCI Timespan=All years

Edit

#6 100,679

#5 OR #4

Indexes=SCI-EXPANDED, SSCI, A&HCI, CPCI-S, CPCI-SSH, ESCI Timespan=All years

Edit

#5 53,868

TI= (mesenchym* stem cell*) OR TI= (stroma* cells*) OR TI= (Multipotent Stem Cell*)

Indexes=SCI-EXPANDED, SSCI, A&HCI, CPCI-S, CPCI-SSH, ESCI Timespan=All years

Edit

#4 90,398

TS=("Mesenchymal Stem Cell*") OR TS=("Mesenchymal Stem Cell Transplantation") OR TS=("Multipotent Stem Cell*") OR TS=("Mesenchymal Stromal Cell*") OR TS=("marrow stroma* cell*")

Indexes=SCI-EXPANDED, SSCI, A&HCI, CPCI-S, CPCI-SSH, ESCI Timespan=All years

Edit

#3 105,966

#2 OR #1

Indexes=SCI-EXPANDED, SSCI, A&HCI, CPCI-S, CPCI-SSH, ESCI Timespan=All years

Edit

#2 22,849

TI=(fresh* thaw*) OR TI=(freez* AND thaw*) OR TI=(cryopreserv*) OR TI=(cryoconserv*)

Indexes=SCI-EXPANDED, SSCI, A&HCI, CPCI-S, CPCI-SSH, ESCI Timespan=All years

Edit

#1 105,775

TS=(Cryopreserv*) OR TS=(cryoconserv*) OR TS=(fresh* NEAR/3 thaw*) OR TS=(freez* NEAR/3 thaw*) OR TS=(fresh cell*) OR TS=(continuously cultur*) OR TS=(fresh* NEAR/3 cultur)

Indexes=SCI-EXPANDED, SSCI, A&HCI, CPCI-S, CPCI-SSH, ESCI Timespan=All years

Embase Classic+Embase <1947 to **2022 January 13**>

Ovid MEDLINE(R) ALL <1946 to **January 13, 2022>**

1 Mesenchymal Stromal Cells/ 56498

2 Mesenchymal Stem Cell Transplantation/ 27041

3 Multipotent Stem Cells/ 9219

4 (mesenchymal adj3 (cell* or stem or stromal or progenitor or multipotent or bone marrow or adipose or placenta*)).tw. 191597

5 ((multipotent or multi-potent) adj (stroma* cell* or stem cell* or progenitor cell*)).tw,kw. 6376

6 (marrow stroma* adj2 cell*).tw,kw. 18764

7 (mesenchymal or marrow strom* or strom* cell*).kw,kw. 4895

8 or/1-7 229245

9 exp Cryopreservation/ 86132

10 cryoconserv*.tw,kw. 585

11 cryopreserv*.tw,kw. 66035

12 (fresh* adj5 thaw*).tw. 4707

13 (freez* adj3 thaw*).tw. 30055

14 (fresh cell* or continuously cultur*).tw,kw. 3007

15 (thaw* or freez*).kw. 13487

16 (fresh* adj3 cultur*).tw. 8848

17 or/9-16 153889

18 8 and 17 3157

19 18 use medall 1180

20 limit 19 to dt=20200601-20220114 146

21 exp mesenchymal stem cell/ 105731

22 mesenchymal stem cell transplantation/ 27041

23 mesenchymal stroma cell/ 15056

24 (mesenchymal adj3 (cell* or stem or stromal or progenitor or multipotent or bone marrow or adipose or placenta*)).tw. 191597

25 ((multipotent or multi-potent) adj (stroma* cell* or stem cell* or progenitor cell*)).tw. 6351

26 (marrow stroma* adj2 cell*).tw. 18733

27 or/21-26 230835

28 cryopreservation/ 72559

29 cryopreserv*.tw. 64484

30 (freez* adj3 thaw*).tw. 30055

31 cryoconserv*.tw. 547

32 (fresh* adj5 thaw*).tw. 4707

33 (fresh* adj3 cultur*).tw. 8848

34 fresh cell*.tw. 2269

35 continuously cultured.tw. 694

36 or/28-35 130925

37 27 and 36 3056

38 conference abstract.pt. 4297058

39 37 not 38 2459

40 39 use emczd 1428

41 **limit 40 to dc=20200601-20220114** 253

42 20 or 41 399

43 remove duplicates from 42 284

**Web of Science – January 14, 2022**

1. TS=(Cryopreserv*) OR TS=(cryoconserv*) OR TS=(fresh* NEAR/3 thaw*) OR TS=(freez* NEAR/3 thaw*) OR TS=(fresh cell*) OR TS=(continuously cultur*) OR TS=(fresh* NEAR/3 cultur)

2. TI=(fresh* thaw*) OR TI=(freez* AND thaw*) OR TI=(cryopreserv*) OR TI=(cryoconserv*)

3. #2 OR #1

4. TS=("Mesenchymal Stem Cell*") OR TS=("Mesenchymal Stem Cell Transplantation") OR TS=("Multipotent Stem Cell*") OR TS=("Mesenchymal Stromal Cell*") OR TS=("marrow stroma* cell*")

5. TI= (mesenchym* stem cell*) OR TI= (stroma* cells*) OR TI= (Multipotent Stem Cell*)

6. #4 OR #5

7. #6 AND #3

8. #6 AND #3 and Meeting Abstracts (Exclude – Document Types)

**Limit to 20200601-20220114 (238)**
