# Supplementary material for: Comparison of freshly cultured versus cryopreserved mesenchymal stem cells in animal models of inflammation: A pre-clinical systematic review
Source: eLife. 2022 Jul 15;11:e75053. doi: 10.7554/eLife.75053 (PMC9286731; doi:10.7554/eLife.75053)
Supplement: Supplementary file 3. [file elife-75053-supp3.docx]

| *The additional signaling questions are included to assist judgment. “Yes” indicates low risk of bias; “no” indicates high risk of bias; and “unclear” indicates an unclear risk of bias. If one of the relevant signaling questions is answered with “no,” this indicates high risk of bias for that specific entry.* | |
| --- | --- |
| **1) Was the allocation sequence adequately generated and applied?** | |
| *Did the investigators describe a random component in the sequence generation process such as: | Yes/No/Unclear |
| ■ Referring to a random number table; |  |
| ■ Using a computer random number generator. |  |
| Additional info: |  |
| Examples of a non-random approach: |  |
| ■ Allocation by judgment or by investigator’s preference; |  |
| ■ Allocation based on the results of a laboratory test or a series of tests; |  |
| ■ Allocation by availability of the intervention; |  |
| ■ Sequence generated by odd or even date of birth; |  |
| ■ Sequence generated by some rule based on animal number or cage number. |  |
| **2) Were the groups similar at baseline or were they adjusted for confounders in the analysis?** |  |
| *Was the distribution of relevant baseline characteristics balanced for the intervention and control groups? | Yes/No/Unclear |
| *If relevant, did the investigators adequately adjust for unequal distribution of some relevant baseline characteristics in the analysis? | Yes/No/Unclear |
| *Was the timing of disease induction adequate? | Yes/No/Unclear |
| Additional info: |  |
| The number and type of baseline characteristics are dependent on the review question. Before starting their risk of bias assessment, therefore, reviewers need to discuss which baseline characteristics need to be comparable between the groups. In an SR investigating the effects of hypothermia on infarct size, for example, gender distribution, left ventricular weight and heart rate and blood pressure should be similar between the groups at the start of the study. |  |
| A description of baseline characteristics and/or confounders usually contains: |  |
| ■ The sex, age and weight of the animals |  |
| ■ Baseline values of the outcomes which are of interest in the study |  |
| Timing of disease induction: |  |
| In some prevention studies, the disease is induced after allocation of the intervention. For example, in an experiment on preventive probiotic supplementation in acute pancreatitis, pancreatitis is induced after allocation of the animals to the probiotic or control group. To reduce baseline imbalance, the timing of disease induction should be equal for both treatment groups. |  |
| Examples of adequate timing of disease induction: |  |
| ■ The disease was induced before randomization of the intervention. |  |
| ■ The disease was induced after randomization of the intervention, but the timing of disease induction was at random, and the individual inducing the disease was adequately blinded from knowing which intervention each animal received. |  |
| **3) Was the allocation to the different groups adequately concealed during?** |  |
| *Could the investigator allocating the animals to intervention or control group not foresee assignment due to one of the following or equivalent methods? | Yes/No/Unclear |
| ■ Third-party coding of experimental and control group allocation Central randomization by a third party |  |
| Sequentially numbered opaque, sealed envelopes |  |
| Additional info: |  |
| Examples of investigators allocating the animals being possibly able to foresee assignments: |  |
| ■ Open randomization schedule |  |
| ■ Envelopes without appropriate safeguard |  |
| ■ Alternation or rotation |  |
| ■ Allocation based on date of birth |  |
| ■ Allocation based on animal number |  |
| ■ Any other explicitly unconcealed procedure of a non-random approach |  |
| **4) Were the animals randomly housed during the experiment?** |  |
| *Did the authors randomly place the cages or animals within the animal room/facility? | Yes/No/Unclear |
| ■ Animals were selected at random during outcome assessment (use signaling questions of entry 6). |  |
| *Is it unlikely that the outcome or the outcome measurement was influenced by not randomly housing the animals? | Yes/No/Unclear |
| The animals from the various experimental groups live together in one cage/pasture (e.g., housing conditions are identical). |  |
| Additional info: |  |
| Examples of investigators using a non-random approach when placing the cages: |  |
| ■ Experimental groups were studied on various locations (e.g., group A in lab A or on shelf A; Group B in Lab B or on shelf B). |  |
| **5) Were the caregivers and/or investigators blinded from knowledge which intervention each animal received during the experiment?** |  |
| *Was blinding of caregivers and investigators ensured, and was it unlikely that their blinding could have been broken? | Yes/No/Unclear |
| ■ ID cards of individual animals, or cage/animal labels are coded and identical in appearance. |  |
| ■ Sequentially numbered drug containers are identical in appearance. |  |
| ■ The circumstances during the intervention are specified and similar in both groups (#). |  |
| ■ Housing conditions of the animals during the experiment are randomized within the room (use criteria of entry 4). |  |
| Additional info: |  |
| Examples of inappropriate blinding: |  |
| ■ Colored cage labels (red for group A, yellow group B) |  |
| ■ Expected differences in visible effects between control and experimental groups |  |
| ■ Housing conditions of the animals are not randomized within the room during the experiment; use criteria of entry 4 |  |
| ■ The individual who prepares the experiment is the same as the one who conducts and analyses the experiment |  |
| ■ Circumstances during the intervention are not similar in both groups (#) |  |
| Examples where circumstances during the intervention were not similar: |  |
| ■ Timing of administration of the placebo and exp drug was different. |  |
| ■ Instruments used to conduct experiment differ between experimental and control group (e.g., experiment about effects abdominal pressure; exp group receives operation and needle to increase pressure, while control group only has the operation). |  |
| ***The relevance of the above-mentioned items depends on the experiment. Authors of the review need to judge for themselves which of the above-mentioned items could cause bias in the results when not similar. These should be assessed.* |  |
| **6) Were animals selected at random for outcome assessment?** |  |
| *Did the investigators randomly pick an animal during outcome assessment, or did they use a random component in the sequence generation for outcome assessment? | Yes/No/Unclear |
| ■ Referring to a random number table; |  |
| ■ Using a computer random number generator; |  |
| ■ Etc. |  |
| **7) Was the outcome assessor blinded?** |  |
| *Was blinding of the outcome assessor ensured, and was it unlikely that blinding could have been broken? | Yes/No/Unclear |
| ■ Outcome assessment methods were the same in both groups. |  |
| ■ Animals were selected at random during outcome assessment (use signaling questions of entry 6). |  |
| *Was the outcome assessor not blinded, but do review authors judge that the outcome is not likely to be influenced by lack of blinding? | Yes/No/Unclear |
| (e.g., mortality) |  |
| Additional info: |  |
| This item needs to be assessed for each main outcome. |  |
| **8) Were incomplete outcome data adequately addressed? (*)** |  |
| *Were all animals included in the analysis? | Yes/No/Unclear |
| *Were the reasons for missing outcome data unlikely to be related to true outcome? (e.g., technical failure) | Yes/No/Unclear |
| *Are missing outcome data balanced in numbers across intervention groups, with similar reasons for missing data across groups? | Yes/No/Unclear |
| *Are missing outcome data imputed using appropriate methods? | Yes/No/Unclear |
| **9) Are reports of the study free of selective outcome reporting? (*)** |  |
| *Was the study protocol available and were all of the study’s pre-specified primary and secondary outcomes reported in the current manuscript? | Yes/No/Unclear |
| *Was the study protocol not available, but was it clear that the published report included all expected outcomes (i.e. comparing methods and results section)? | Yes/No/Unclear |
| Additional info: |  |
| Selective outcome reporting: |  |
| - Not all of the study’s pre-specified primary outcomes have been reported; |  |
| - One or more primary outcomes have been reported using measurements, analysis methods or data subsets (e.g., subscales) that were not pre-specified in the protocol; |  |
| - One or more reported primary outcomes were not pre-specified (unless clear justification for their reporting has been provided, such as an unexpected adverse effect); |  |
| - The study report fails to include results for a key outcome that would be expected to have been reported for such a study. |  |
| **10) Was the study apparently free of other problems that could result in high risk of bias? (*)** |  |
| *Was the study free of contamination (pooling drugs)? | Yes/No/Unclear |
| *Was the study free of inappropriate influence of funders? | Yes/No/Unclear |
| *Was the study free of unit of analysis errors? | Yes/No/Unclear |
| *Were design-specific risks of bias absent? | Yes/No/Unclear |
| *Were new animals added to the control and experimental groups to replace drop-outs from the original population? | Yes/No/Unclear |
| Additional info: |  |
| The relevance of the signaling questions (Table [3](https://bmcmedresmethodol.biomedcentral.com/articles/10.1186/1471-2288-14-43#Tab3)) depends on the experiment. Review authors need to judge for themselves which of the items could cause bias in their results and should be assessed. |  |
| Contamination/pooling drugs: |  |
| Experiments in which animals receive ‒ besides the intervention drug ‒ additional treatment or drugs which might influence or bias the result. |  |
| Unit of analysis errors: |  |
| ■ Interventions to parts of the body within one participant (i. e., one eye exp; one eye control). |  |
| ■ All animals receiving the same intervention are caged together, but analysis was conducted as if every single animal was one experimental unit. |  |
| Design-specific risks of bias: |  |
| ■ Crossover design that was not suitable (intervention with no temporary effect, or the disease is not stable over time) |  |
| ■ Crossover design with risk of carry-over effect |  |
| ■ Crossover design with only first period data being available |  |
| ■ Crossover design with many animals not receiving 2^nd^ or following treatment due to large number of drop-outs probably due to longer duration of study |  |
| ■ Crossover design in which all animals received same order of interventions |  |
| ■ Multi-arm study in which the same comparisons of groups are not reported for all outcomes (selective outcome reporting) |  |
| ■ Multi-arm study in which results of different arms are combined (all data should be presented per group) |  |
| ■ Cluster randomized trial not taking clustering into account during statistical analysis (unit of analysis error) |  |
| ■ Crossover design in which paired analysis of the results is not taken into account |  |
