# Supplementary material for: Comparison of freshly cultured versus cryopreserved mesenchymal stem cells in animal models of inflammation: A pre-clinical systematic review
Source: eLife. 2022 Jul 15;11:e75053. doi: 10.7554/eLife.75053 (PMC9286731; doi:10.7554/eLife.75053)
Supplement: Supplementary file 4. [file elife-75053-supp4.docx]

| Category | Specific Items |
| --- | --- |
| Study characteristics | Study title, author, date of publication,  journal published, country of publication, randomization process |
| Study population  (animal model) | Animal type, age, gender, strain, and  weight, presence of co-morbid illnesses |
| Model of Inflammation and method of induction | Acute or chronic, Direct or indirect infection,  Chemical-induced injury, ischemia-reperfusion, surgically-induced model, details of model induction process |
| Intervention and comparison | International Society for Cellular Therapy criteria, MSC tissue source, MSC characteristics (including method of sorting), time and route of MSC administration, description of preparation and suspension of MSCs and controls (including medium used for cells, duration of culture, passage number, concentration/amount of MSCs used), vehicle used for delivery, storage conditions, cryopreservation methods (duration of cryopreservation, method used to cryopreserved MSCs, and concentration of MSCs during cryopreservation, and medium used to cryopreserve and whether any additives were used), post-thaw washing procedure and time to use in experiment, time from disease induction to MSC administration, MSC viability, surface marker expression |
| Co-interventions | Antibiotics, cytokines, cultured medium, membrane/scaffold system employed |
| Potency | MSC secretome and release assay, MSC effect on various cells, and PBMC proliferation co-culture assay |
| Surrogate Markers of Efficacy | Preclinical outcomes that are relevant as per the model of inflammation employed and fall within the two domains of Organ Dysfunction and Protein Expression and Secretion: examples include arterial oxygenation, lung compliance, neovascularization, epithelialization, wound contraction rate, arthritic index, histological lung injury, etc. |
| Other | Presence of *a priori* sample size calculation.  Industry sponsorship |

**Supplementary Table 2.** Details of the data collection domains included in our standardized excel-sheet for data extraction is presented here.
